# Milk fat globule—EGF factor 8/ATP‐binding cassette subfamily E member 1 axis maintains mitophagy flux homeostasis to suppress ferroptosis in acute pancreatitis

**DOI:** 10.1002/ctm2.70619

**Published:** 2026-02-18

**Authors:** Yifan Ren, Yuxuan Lu, Qing Cui, Hao Shang, Meng Fan, Yun Sun, Xiali Shi, Rongqian Wu, Hongwei Lu

**Affiliations:** ^1^ Department of General Surgery The Second Affiliated Hospital of Xi'an Jiaotong University Xi'an China; ^2^ National Local Joint Engineering Research Center for Precision Surgery & Regenerative Medicine Shaanxi Provincial Center for Regenerative Medicine and Surgical Engineering First Affiliated Hospital of Xi'an Jiaotong University Xi'an China; ^3^ Department of Hepatobiliary Surgery, Beijing Tsinghua Changgung Hospital, School of Clinical Medicine Tsinghua University Beijing China; ^4^ Department of Cardiology Xi'an Third Hospital Affiliated to Northwest University Xi'an China; ^5^ Department of Anesthesiology and Surgery The Second Affiliated Hospital of Xi'an Jiaotong University Xi'an China

**Keywords:** ABCE1, acute pancreatitis, ferroptosis, MFG‐E8, mitophagy

## Abstract

**Background:**

Acute pancreatitis (AP) is a severe inflammatory disorder in which mitochondrial dysfunction and ferroptosis critically drive acinar cell injury. Our previous work suggested a protective role for exogenous milk fat globule—epidermal growth factor 8 (MFG‐E8) in AP. This study aimed to elucidate the molecular mechanism by which endogenous MFG‐E8 mitigates mitochondrial damage and ferroptosis during AP.

**Methods:**

Two mouse models of AP were used for in vivo studies, while cerulein + lipopolysaccharide‐induced mitophagy and ferroptosis in AR42J cells (cells of the rat exocrine pancreas) for in vitro studies. *Mfge8* gene‐defective mice and lentivirus were utilised to downregulate MFG‐E8 expression in mice and overexpress MFG‐E8 in cells, respectively. Dual gene modification was employed to overexpress MFG‐E8 and simultaneously knockdown adenosine triphosphate (ATP)‐binding cassette subfamily E member 1 (ABCE1) in vitro. One mitophagy agonist and two ferroptosis inhibitors were used in both in vitro and in vivo experiments.

**Results:**

Endogenous MFG‐E8 expression was downregulated in experimental AP. Genetic deletion of *Mfge8* aggravated mitochondrial ultrastructural damage, impaired mitophagy flux and intensified ferroptosis, as evidenced by increased lipid peroxidation, Fe^2+^ accumulation and depletion of glutathione peroxidase. Lentiviral overexpression of MFG‐E8 in AR42J acinar cells restored mitophagy activity, preserved mitochondrial membrane potential and reduced oxidative stress. Mechanistically, co‐immunoprecipitation confirmed that MFG‐E8 directly interacts with ABCE1, a key mitophagy regulator. ABCE1 knockdown abolished the protective effects of MFG‐E8 on mitochondrial function and ferroptosis suppression, indicating that the MFG‐E8/ABCE1 axis is essential for maintaining mitophagy homeostasis. Pharmacological restoration of mitophagy or inhibition of ferroptosis rescued acinar cell injury caused by MFG‐E8/ABCE1 dysregulation. In vivo, ferroptosis inhibition significantly improved pancreatic pathology and survival in *Mfge8*‐deficient AP mice.

**Conclusion:**

Endogenous MFG‐E8 protects against AP by binding ABCE1 to sustain mitophagy flux and inhibit ferroptosis. Targeting this axis offers a promising therapeutic strategy for mitigating pancreatic injury.

**Key points:**

- Endogenous MFG‐E8 is downregulated in acute pancreatitis (AP), disrupting MFG‐E8/ABCE1 complex formation.
- MFG‐E8/ABCE1 axis sustains Parkin‐PINK1‐mediated mitophagy to clear damaged mitochondria in pancreatic acinar cells.
- This axis suppresses ferroptosis by reducing Fe^2+^ accumulation and lipid peroxidation, alleviating AP‐related pancreatic injury.

## Graphical abstract

During AP, MFG‐E8 expression in pancreatic acinar cells is downregulated, resulting in disruption of the ABCE1/MFG‐E8 complex. This impairment leads to defective mitophagy flux, accumulation of lipid peroxides, endoplasmic reticulum stress and ferroptosis, ultimately contributing to pancreatic damage

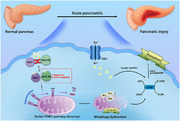

## 1 INTRODUCTION

Acute pancreatitis (AP) is a life‐threatening inflammatory disease characterised by premature activation of pancreatic enzymes, leading to autodigestion, pancreatic injury and systemic inflammatory response syndrome.^1^ In severe cases, AP can progress to multi‐organ failure, significantly increasing morbidity and mortality.^2^ Emerging evidence highlights that mitophagy dysfunction acts as a pivotal link between AP pathogenesis and the induction of ferroptosis, a distinct form of regulated cell death driven by iron‐dependent lipid peroxidation.^3^, ^4^ The intricate interplay among AP, mitophagy dysregulation and ferroptosis not only deepens our understanding of the disease's pathophysiology but also provides potential therapeutic avenues for interrupting this detrimental cycle and improving patient prognosis.

Milk fat globule—epidermal growth factor 8 (MFG‐E8) is a multifunctional glycoprotein with wide tissue expression.^5^, ^6^ It acts as a bridging molecule mediating apoptotic cells and phagocytes, facilitating efferocytosis to preserve tissue homeostasis and mitigate inflammatory responses.^7^, ^8^ Beyond these functions, accumulating evidence highlights its critical involvement in tissue degeneration and regeneration—for instance, its downregulation in degenerative intervertebral disc (IVD) tissues correlates with chondrocyte apoptosis and extracellular matrix (ECM) degradation, while exogenous MFG‐E8 supplementation promotes IVD cell survival and ECM synthesis. Its regenerative potential has been harnessed in biomaterials‐mediated therapy, where MFG‐E8‐loaded hydrogels or extracellular vesicles enable sustained local delivery, enhancing IVD regeneration in preclinical models.^9^ Our previous study demonstrated that intraperitoneal injection of exogenous MFG‐E8 participates in alleviating mitochondrial dysfunction in pancreatic acinar of AP mice by activating the FAK‒STAT3 pathway via integrin αVβ3/5 receptors.^10^ In that study, we found that eliminating the expression of endogenous MFG‐E8 by knocking out the *mfge8* gene aggravated mitochondrial dysfunction in AP mice and worsened pancreatic damage. Therefore, we hypothesise whether endogenous MFG‐E8 also protects the mitochondrial dysfunction caused by AP in some way.

Adenosine triphosphate (ATP)‐binding cassette subfamily E member 1 (ABCE1), an ATP‐dependent transporter, is essential for ribosome biogenesis and cellular redox balance.^11^, ^12^ Recent studies show its role in regulating mitophagy, the process of interacting with key mitophagy regulators (e.g., PINK1‒Parkin pathway) to ensure efficient mitochondrial clearance.^13^ Meanwhile, ABCE1 plays a crucial role in the mitochondrial quality control and translation regulation of axons.^14^ To search for relevant evidence that MFG‐E8 might be involved in the pathogenesis of AP by regulating mitophagy, we screened in the BioGRID database and found that MFG‐E8 could combine with ABCE1. Therefore, the question of whether endogenous MFG‐E8 regulates mitophagy by binding to ABCE1 and thereby promotes the recovery of damaged pancreatic acinar cells in AP naturally emerges on the surface. This study aims to clarify the relationship between endogenous MFG‐E8 and mitophagy dysfunction as well as the resulting cell death in AP.

## 2 MATERIALS AND METHODS

### 2.1 Patients

A total of 85 adult patients with AP (aged ≥18 years) who were hospitalised at the First Affiliated Hospital of Xi'an Jiaotong University (XJTU) were enrolled in the present study. The diagnosis of AP was made in accordance with the criteria established by the 2012 International Atlanta Symposium on AP.^15^ Disease severity for all AP patients was evaluated utilising the RANSON score and the BISAP score. This research protocol was granted ethical approval by the Ethics Committee of the First Affiliated Hospital of XJTU. Informed consent was obtained from all participants, in strict adherence to the principles outlined in the Declaration of Helsinki.

### 2.2 Animals and gene editing

For the animal experiments, 8‒10‐week‐old male C57BL/6J mice were employed, sourced from Huaren Biotechnology Co., Ltd. The *mfge8* gene knockout (*mfge8*‐KO) mice were created through the CRISPR/Cas9 method (Shanghai Model Organisms Center, Inc.), which involved the deletion of exons 2‒6 of the *mfge8* gene on a C57BL/6J genetic background. Meanwhile, wild‐type (WT) littermates served as the control group. Mice were euthanised via isoflurane inhalation anaesthesia (5% induction and 2% maintenance) followed by cervical dislocation, which was performed in accordance with the Guidelines for the Euthanasia of Animals from the American Veterinary Medical Association to ensure minimal pain and distress.

### 2.3 Animal models

Arginine‐induced AP models were established in mice via intraperitoneal injection of L‐arginine (4 g/kg, cat. no. A640158, Aladdin Scientific) at 2 h intervals.^16^ At 72 h after the first injection, mice were anaesthetised with isoflurane inhalation, and blood samples plus pancreatic tissues were collected. For a separate L‐arginine‐induced AP cohort, normal saline (vehicle control) was administered at 3 h after the initial L‐arginine injection (vehicle) or  .2,  .4,  .6,  .8 and 1.0 mg/kg Fer‐1 (S7243, Selleck, Inc.) was administered. Mice were anaesthetised at 72 h after the initial L‐arginine injection (i.e., 69 h following Fer‐1 administration). For separate cohorts of L‐arginine‐induced AP mice, survival rates were continuously monitored over a 5‐day period after treatment with vehicle or Fer‐1.

Cerulein + lipopolysaccharide (LPS)‐induced AP was induced in mice via seven intraperitoneal (i.p.) injections of cerulein (50 µg/kg, cat. no. C6660, Solarbio) administered at 1‐h intervals, with LPS (10 mg/kg, cat. no. L8880, Solarbio) co‐administered in the final cerulein injection.^10^ Mice were anaesthetised at 11 h after the initial cerulein injection (i.e., 4 h after the final cerulein administration). For separate cohorts of cerulein/LPS‐induced AP mice, survival was continuously monitored over a 5‐day observational period subsequent to treatment with vehicle or Fer‐1.

### 2.4 Cell culture and treatments

Pancreatic AR42J cells (cells of the rat exocrine pancreas) (cat. no. CL‐0025, Procell Life Science) were maintained in a cell‐type‐specific medium for AR42J cells (cat. no. CM‐0025, Procell Life Science) under a standard humidified incubational condition (37°C, 5% CO_2_).^10^ The cells were seeded into six‐well culture plates or laser confocal dishes at a density of 5 × 10^5^ cells per well for subsequent experimental assays. Prior to experimental manipulation, AR42J cells were pretreated with 100 nM dexamethasone (cat. no. D4902, Sigma‒Aldrich) for 48 h to induce their activation and differentiation into acinar‐like phenotypic characteristics.

AR42J cells were exposed to a combination of cerulein (100 nmol/L, cat. no. C6660, Solarbio) and LPS (10 ng/mL, cat. no. L‐8880, Solarbio) for a 24‐h incubation period. Cells in the control group received an equal volume of the complete culture medium alone. Lentiviral vectors encoding MFG‐E8 (Lv‐MFG‐E8, overexpression construct) and the empty lentiviral vector (Lv‐vector, negative control) were custom‐synthesised by Soochow Cyagen Biosciences Co., Ltd. Lentiviral transfection was subsequently performed in accordance with the manufacturer's standard operating protocols.

AR42J cells were treated with cerulein and LPS for 24 h. The AR42J cell line with *Abce1* knockout (*abce1*‐KO) was constructed using CRISPR/Cas9 gene‐editing technology. On the basis of this cell line, we further transfected it with MFG‐E8‐overexpressing lentivirus to achieve the overexpression of *mfge8* gene in *abce1*‐KO AR42J monoclonal cells (Lv‐*mfge8* + *abce1*‐KO, dual genetic modification, Soochow Cyagen Corporation).

In another experiment, AR42J cells were treated with cerulein and LPS for 24 h. A specific mitophagy agonist, mitochonic acid 5 (MA‐5; 0, 20, 50, 100 or 150 µM; S0881, Selleck, Inc.) were added into AR42J that overexpressed *mfge8* with *abce1*‐KO.

In another experiment, AR42J cells were treated with cerulein and LPS for 24 h. Two specific ferroptosis inhibitors, Ferrostatin‐1 (Fer‐1; 0, 1, 5, 10 or 20 µM; S7243, Selleck, Inc.) and Liproxstatin‐1 (Lip‐1; 0, 20, 30, 40 or 60 nM; S7699, Selleck, Inc.), were added into AR42J that overexpressed *mfge8* with *abce1*‐KO.

The methods for haematoxylin and eosin, immunofluorescence staining, transmission electron microscopy, Mito‐Tracker and Mito‐SOX staining, FerroOrange and BODIPY 581/591 staining, enzyme‐linked immunosorbent assays, western blot analysis, biochemical analysis, co‐immunoprecipitation (Co‐IP), statistical analyses and the construction reports of the stable cell lines are provided in the Supporting Information.

## 3 RESULTS

### 3.1 MFG‐E8 deficiency aggravated ferroptosis and deteriorated mitophagy in experimental AP

**FIGURE 1:**
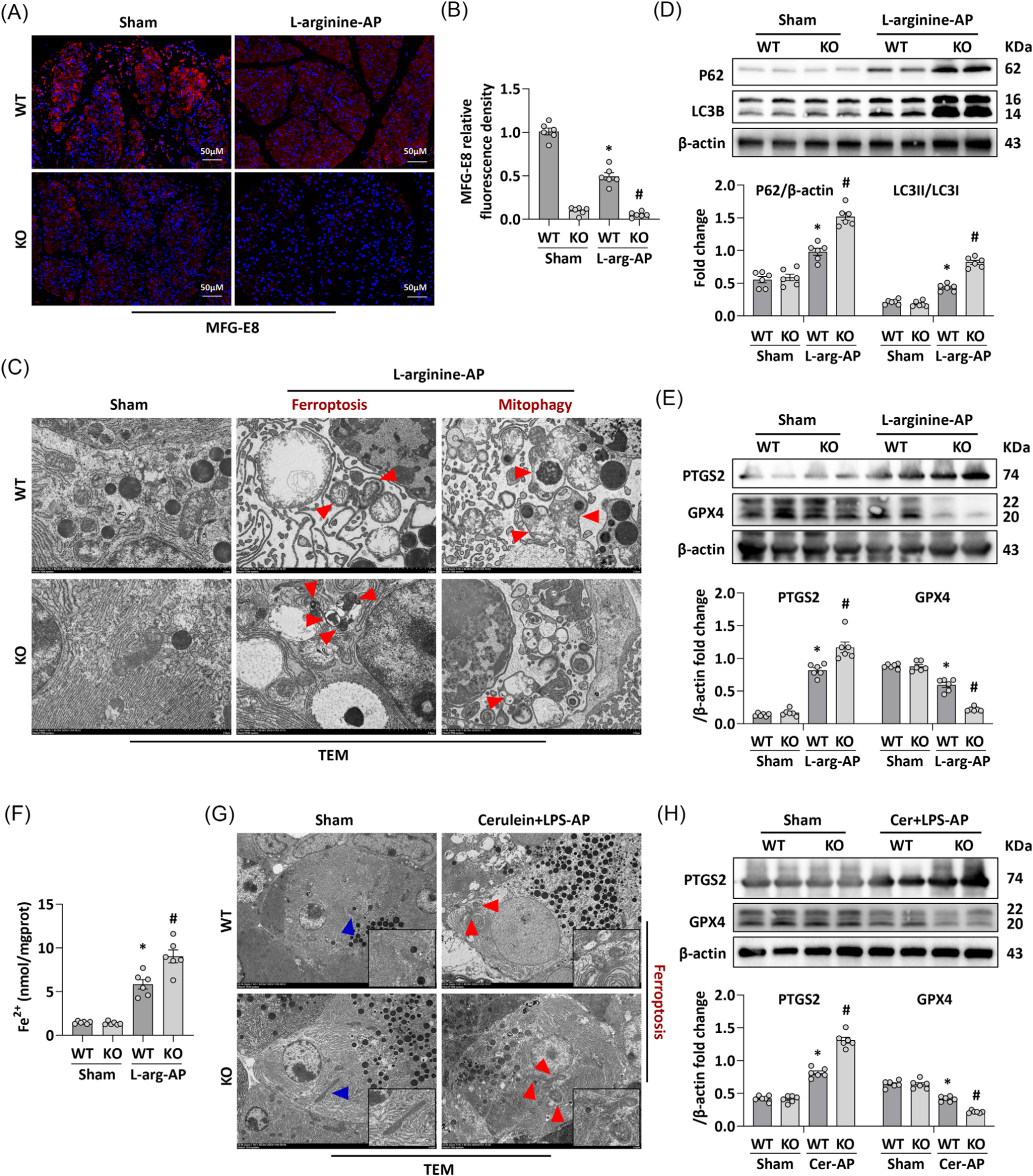
Milk fat globule—epidermal growth factor 8 (MFG‐E8) deficiency aggravated ferroptosis and deteriorated mitophagy in experimental acute pancreatitis (AP). (A) Representative images of MFG‐E8 staining (200×). (B) Quantitative of MFG‐E8 staining. (C) Ultrastructural alterations in the pancreas. (D) Western blot analysis of the P62 and LC3B expression level in the pancreas. (E) Western blot analysis of the Prostaglandin‐Endoperoxide Synthase 2 (PTGS2) and glutathione peroxidase (GPX4) expression level in the pancreas. (F) Fe^2+^ levels in the pancreas. (G) Ultrastructural alterations in the pancreas. (H) Western blot analysis of the PTGS2 and GPX4 expression level in the pancreas. *n* = 6, error bars indicate the SEM; ^*^ *p* < .05 versus wild type (WT)‐Sham; ^#^ *p* < .05 versus WT‐AP. KO, knockout; LPS, lipopolysaccharide; TEM, transmission electron microscopy.

Our previous findings revealed that intraperitoneal administration of exogenous MFG‐E8 exerted a reparative effect on mitochondrial dysfunction in AP‐induced mice. To further investigate the role of endogenous MFG‐E8 in AP pathogenesis, we generated *Mfge8*‐KO mice and established an in vivo AP model. As illustrated in Figure 1A,B, pancreatic MFG‐E8 expression was significantly downregulated in AP mice, while *Mfge8*‐KO almost completely abrogated MFG‐E8 expression in the pancreas (Figure S1). Ultrastructure revealed that in pancreatic cells of L‐arginine‐induced AP mice, mitochondria exhibited characteristic morphological alterations of ferroptosis, including shrinkage, increased bilayer membrane density, reduction or loss of mitochondrial cristae and outer mitochondrial membrane rupture. Notably, *Mfge8*‐KO further exacerbated these ferroptosis‐associated mitochondrial changes (Figure 1C). Consistent with this, *Mfge8*‐KO led to a decrease in autophagosome formation and aggravated autophagic flux impairment in AP mice, which in turn compromised the cells’ capacity to clear damaged mitochondrial fragments—a process termed mitophagy (Figure 1C). Additionally, the concurrent upregulation of P62 and LC3B protein levels further confirmed that *Mfge8*‐KO exacerbates autophagic dysfunction in pancreatic cells of AP mice (Figure 1D).

Given that oxidative stress is closely intertwined with both mitophagy dysfunction and ferroptosis, we next sought to further confirm ferroptosis induction in AP by assessing the levels of two key ferroptosis‐related proteins (PTGS2 and GPX4) and measuring Fe^2+^ content in pancreatic tissues. As depicted in Figure 1E, L‐arginine‐induced AP led to a modest increase in pancreatic PTGS2 expression and a slight decrease in GPX4 expression in WT mice. In stark contrast, MFG‐E8‐KO mice subjected to L‐arginine‐induced AP exhibited a significant upregulation of PTGS2 and nearly undetectable GPX4 expression (*p* < .05). The anticipated changes were also observed in the Fe^2+^ content. As shown in Figure 1F, Fe^2+^ levels increased in the pancreatic tissues of L‐arginine‐AP mice, and this elevation was exacerbated by *mfge8*‐KO. In addition, we constructed another in vivo model of AP using cerulein + LPS, and confirmed in the changes of mitochondrial ultrastructure and the level of ferroptosis‐related proteins that the deficiency of MFG‐E8 was involved in ferroptosis of AP pancreatic cells caused by different aetiologies (Figure 1G,H).

### 3.2 Overexpression of MFG‐E8 restores mitophagy in the in vitro AP model

**FIGURE 2:**
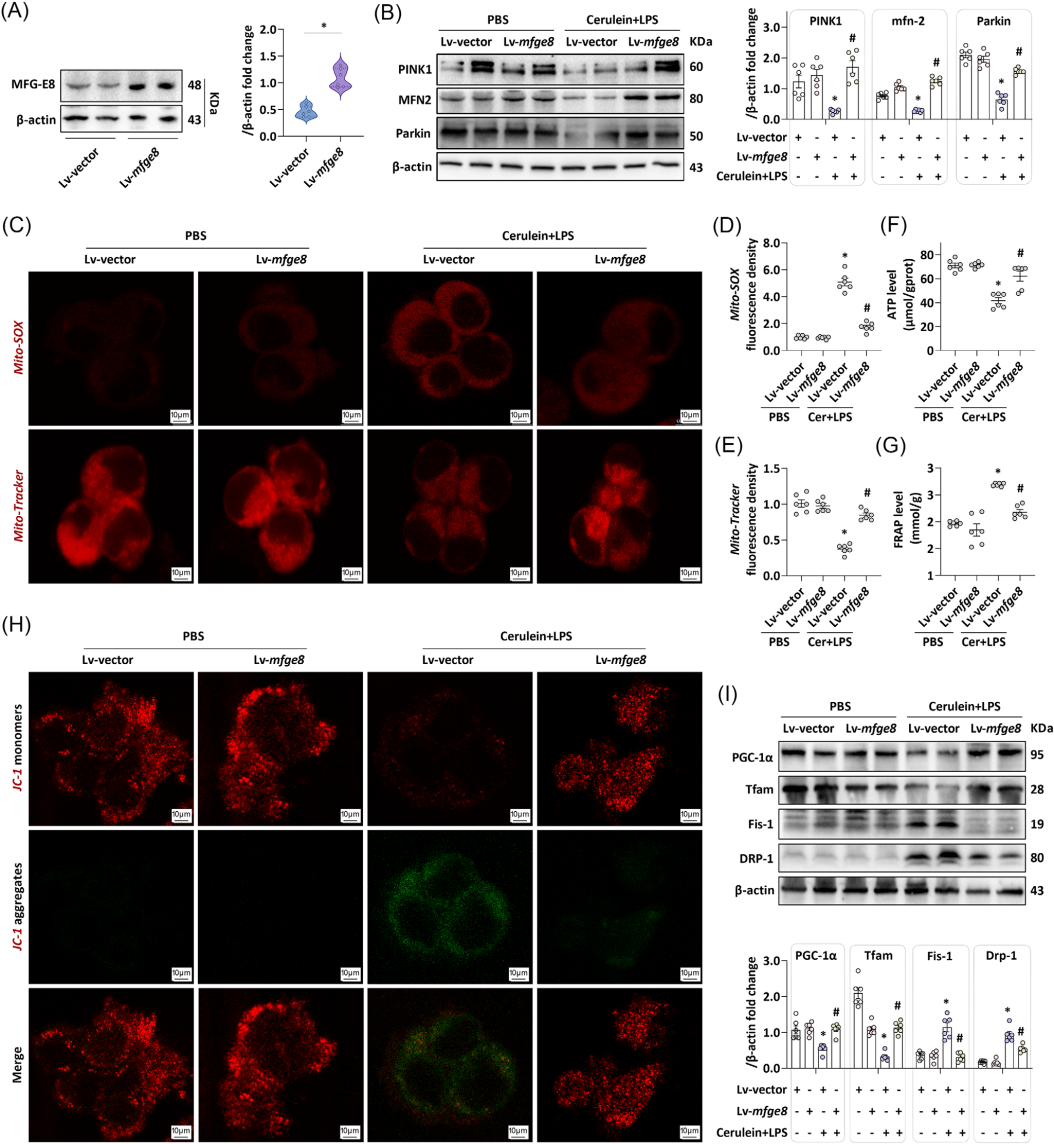
Overexpression of milk fat globule—epidermal growth factor 8 (MFG‐E8) restores mitophagy in experimental acute pancreatitis (AP). (A) Western blot analysis of the MFG‐E8 expression level in AR42J cells. (B) Western blot analysis of the PINK1, Mitofusin 2 (MFN2) and Parkin expression level in AR42J cells. (C) Representative images of Mito‐SOX (1500×) and Mito‐Tracker red (1500×) in AR42J cells. (D and E) Relative fluorescence intensity of Mito‐SOX and Mito‐Tracker red in AR42J cells. (F) Adenosine triphosphate (ATP) levels in AR42J cells. (G) Ferric reducing antioxidant power (FRAP) levels in AR42J cells. (H) Representative images of JC‐1 (1500×) in AR42J cells. (I) Western blot analysis of the peroxisome proliferator‐activated receptor gamma coactivator 1‐alpha (PGC‐1α), mitochondrial transcription factor A (Tfam), fission protein 1 (Fis‐1) and dynamin‐related protein 1 (Drp‐1) expression level in AR42J cells. *n* = 6, error bars indicate the SEM; ^*^ *p* < .05 versus Lv‐vector‐PBS; ^#^ *p* < .05 versus Lv‐vector‐AP. Cer, cerulein; LDH, lactate dehydrogenase; LPS, lipopolysaccharide; Lv, lentivirus.

To elucidate the protective role and underlying mechanism of endogenous MFG‐E8 against AP, we overexpressed MFG‐E8 in AR42J cells (rat pancreatic exocrine acinar cell line) (Figure 2A) and subsequently established an in vitro AP model. As shown in Figure 2B, cerulein + LPS treatment led to downregulation of PINK1—a key regulator of mitophagy—in AR42J cells. Mitofusin‐2 (Mfn2), a downstream effector of PINK1, is phosphorylated in response to PINK1 signalling; this phosphorylation event recruits Parkin to maintain mitophagy function.^17^ Consistent with the reduced PINK1 expression, the protein levels of Mfn2 and Parkin also exhibited a corresponding decrease. Notably, MFG‐E8 overexpression reversed the reductions in PINK1, Mfn2 and Parkin in this in vitro AP model.

To further evaluate mitochondrial oxidative stress, we used Mito‐SOX fluorescent probes to label mitochondrial reactive oxygen species (ROS) and Mito‐Tracker fluorescent probes to label viable mitochondria. As illustrated in Figure 2C‒E, cerulein + LPS‐induced AP in vitro significantly increased Mito‐SOX fluorescence intensity, indicating elevated mitochondrial ROS levels; this aberrant increase was attenuated by MFG‐E8 overexpression. Concurrently, MFG‐E8 overexpression restored the reduced number of viable mitochondria in damaged AR42J cells. Functionally, the aberrant accumulation of damaged mitochondria resulted in mitochondrial dysfunction, which in turn caused impaired ATP synthesis in AR42J cells (Figure 2F). Additionally, ferric ion‐reducing antioxidant power (FRAP) used to assess the mitochondria's capacity to scavenge oxidative metabolites (Figure 2G). MFG‐E8 overexpression restored ATP levels and FRAP activity in the in vitro AP model. A decrease in mitochondrial membrane potential (ΔΨm) is recognised as the earliest hallmark of mitochondrial dysfunction. To directly assess ΔΨm changes in AR42J cells, we utilised JC‐1 fluorescence, which undergoes a characteristic color shift in response to membrane potential alterations.^18^ As shown in Figure 2H, cerulein + LPS treatment induced a JC‐1 fluorescence transition from red to green in AR42J cells, indicative of ΔΨm depolarisation. Consistent with our hypothesis, MFG‐E8 overexpression reversed this depolarisation, restoring normal JC‐1 fluorescence patterns.

Healthy mitochondrial dynamics—encompassing processes such as mitochondrial biogenesis and fission—are indispensable for sustaining proper mitochondrial function. Among the key regulators governing mitochondrial biogenesis, peroxisome proliferator‐activated receptor gamma coactivator 1‐alpha (PGC‐1α) and mitochondrial transcription factor A (Tfam) play pivotal roles.^19^ Western blot analysis revealed that PGC‐1α and Tfam were downregulated in cerulein + LPS‐treated AR42J cell. MFG‐E8 overexpression markedly increased PGC‐1α and Tfam levels in this in vitro AP. For mitochondrial fission, which is regulated by dynamin‐related protein 1 (Drp‐1) and fission protein 1 (Fis‐1),^20^ western blot analysis demonstrated that overexpression of MFG‐E8 downregulated the increased levels of Drp‐1 and Fis‐1 observed in the in vitro AP (Figure 2I). The above results indicate that the increased expression of MFG‐E8 not only restored the mitophagy and ROS of damaged acinar cells, but also promoted the recovery of mitochondrial function. These results also echo the findings of our previous observations,^10^ reinforcing the protective role of MFG‐E8 in maintaining mitochondrial homeostasis during AP.

### 3.3 Overexpression of MFG‐E8 alleviates ferroptosis in the in vitro AP model

**FIGURE 3:**
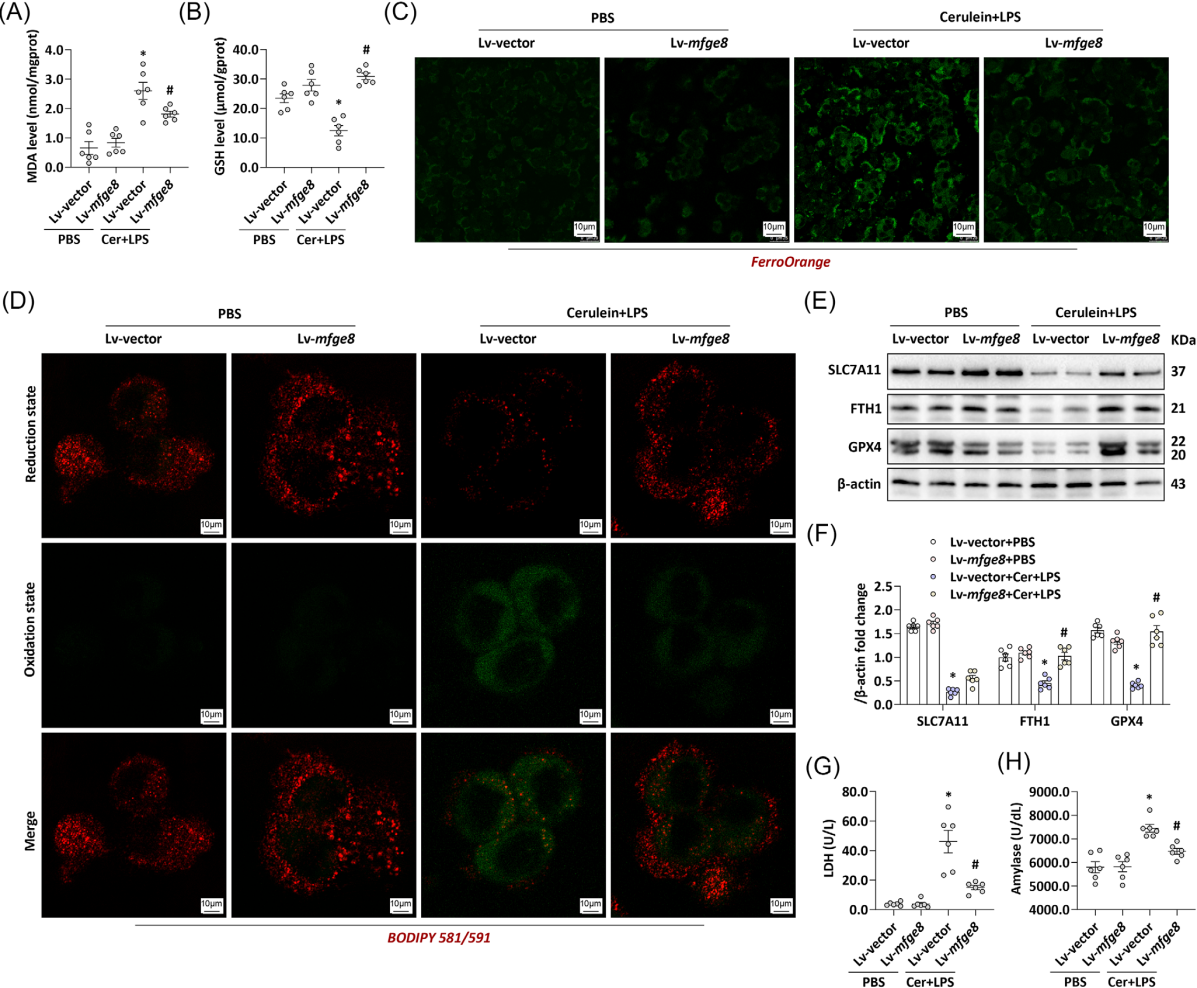
Overexpression of milk fat globule—epidermal growth factor 8 (MFG‐E8) alleviates ferroptosis in experimental acute pancreatitis (AP). (A) Malondialdehyde (MDA) levels in AR42J cells. (B) Glutathione (GSH) levels in AR42J cells. (C) Representative images of FerroOrange (1500×) in AR42J cells. (D) Representative images of BODIPY 581/591 (1500×) in AR42J cells. (E and F) Western blot analysis of the solute carrier family 7 member 11 (SLC7A11), ferritin heavy chain 1 (FTH1) and glutathione peroxidase (GPX4) expression level in AR42J cells. (G) Lactate dehydrogenase (LDH) levels in supernatant. (H) Amylase levels in supernatant. *n* = 6, error bars indicate the SEM; ^*^ *p* < .05 versus Lv‐vector‐PBS; ^#^ *p* < .05 versus Lv‐vector‐AP. Cer, cerulein; LPS, lipopolysaccharide; Lv, lentivirus.

The inhibitory effect of endogenous MFG‐E8 on oxidative stress in the in vitro AP model was also concretised in the expression changes of malondialdehyde and glutathione (GSH), as shown in Figure 3A,B (*p *< .05). ROS accumulation and GSH depletion are recognised as initiating events of ferroptosis, whereas intracellular Fe^2+^ accumulation and lipid peroxidation are hallmark features of this process.^21^ To delineate the role of ferroptosis in this study, we assessed lipid peroxidation levels and intracellular Fe^2+^ content in AR42J cells using fluorescent dyes. As shown in Figure 3C,D, cerulein + LPS treatment increased intracellular Fe^2+^ levels in AR42J (evidenced by enhanced FerroOrange fluorescence) and induced a shift towards lipid peroxidation (reflected by BODIPY 581/591 green fluorescence). These pieces of evidence suggest an increase in intracellular free Fe^2+^ and an intensification of lipid peroxidation. Notably, MFG‐E8 overexpression in AR42J cells abrogated cerulein + LPS‐induced Fe^2+^ accumulation and promoted a shift towards reduced intracellular lipids (evidenced by BODIPY 581/591 red fluorescence).

Solute carrier family 7 member 11 (SLC7A11) primarily functions in regulating GSH metabolism. Inhibition of SLC7A11 and depletion of glutathione peroxidase 4 (GPX4) promote lipid peroxidation, ultimately triggering ferroptosis. As we demonstrated in Figure 3E,F, the in vitro AP model led to the exhaustion of both SLC7A11 and GPX4 in AR42J, accompanied by reduced ferritin heavy chain 1 (FTH1) expression in AR42J cells. Lentivirus‐mediated MFG‐E8 overexpression restored the levels of these ferroptosis‐inhibitory proteins. Concomitantly, MFG‐E8 overexpression attenuated the cerulein + LPS‐induced elevation of lactate dehydrogenase (LDH) and amylase in AR42J cells (*p *< .05, Figure 3G,H), confirming the suppression of ferroptosis.

### 3.4 ABCE1 knockout antagonised the protective role of MFG‐E8 on the in vitro AP

**FIGURE 4:**
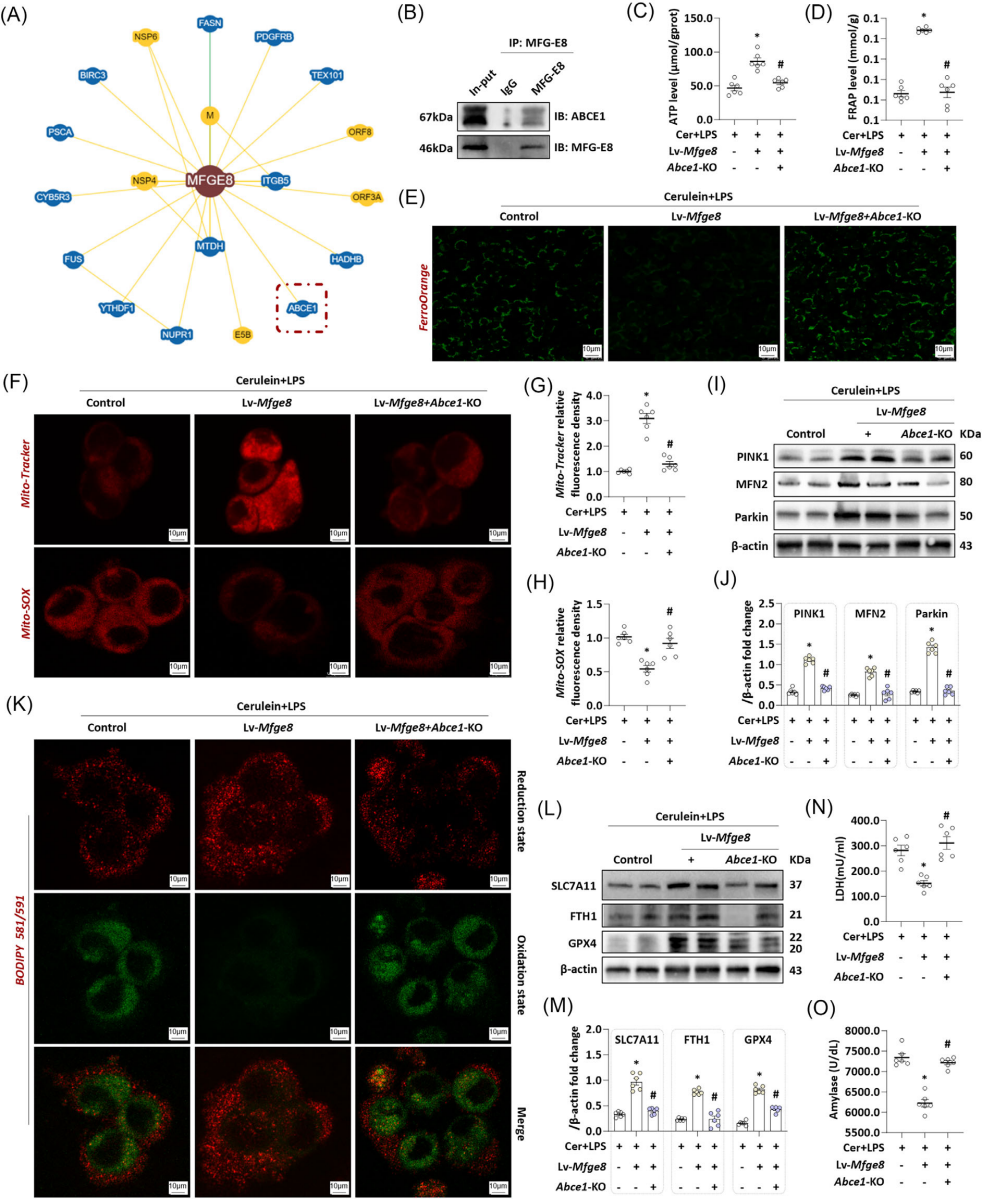
Adenosine triphosphate (ATP)‐binding cassette subfamily E member 1 (ABCE1) knockout antagonised the protective effect of milk fat globule—epidermal growth factor 8 (MFG‐E8) on the in vitro acute pancreatitis (AP). (A) MFG‐E8 directly binds to ABCE1 (BioGRID database). (B) MFG‐E8 directly binds to ABCE1 in AR42J cells. (C) ATP levels in AR42J cells. (D) Ferric reducing antioxidant power (FRAP) levels in AR42J cells. (E) Representative images of FerroOrange (200×) in AR42J cells. (F) Representative images of Mito‐SOX and Mito‐Tracker red (1500×) in AR42J cells. (G and H) Relative fluorescence intensity of Mito‐SOX and Mito‐Tracker red in AR42J cells. (I and J) Western blot analysis of the PINK1, Mitofusin 2 (MFN2) and Parkin expression level in AR42J cells. (K) Representative images of BODIPY 581/591 (1500×) in AR42J cells. (L and M) Western blot analysis of the solute carrier family 7 member 11 (SLC7A11), ferritin heavy chain 1 (FTH1) and glutathione peroxidase (GPX4) expression level in AR42J cells. (N) Lactate dehydrogenase (LDH) levels in supernatant. (O) Amylase levels in supernatant. *n* = 6, error bars indicate the SEM; ^*^ *p* < .05 versus vehicle‐AP (Lv‐vector); ^#^ *p* < .05 versus Lv‐*mfge8*‐AP. Cer, cerulein; IB, immunoblotting; IP, immunoprecipitation; KO, knockout; LPS, lipopolysaccharide; Lv, lentivirus.

To identify potential mediators of MFG‐E8‐dependent mitochondrial function regulation, we queried the BioGRID database and identified ABCE1—a known mitophagy regulator—as a candidate direct binding partner of MFG‐E8 (Figure 4A). This interaction was experimentally validated via Co‐IP, which confirmed that ABCE1 directly binds to MFG‐E8 in AR42J cells (Figure 4B).

To determine whether ABCE1 is required for MFG‐E8‐mediated regulation of mitochondrial function and cellular metabolism in AP, on the basis of overexpressing MFG‐E8, we simultaneously knocked out the ABCE1 gene of AR42J and successfully constructed cells with dual gene modification of *Mfge8* overexpression (*Mfge8*‐OE) + *Abce1*‐KO (Figure S2). As shown in Figure 4C,D, *Abce1*‐KO abrogated the restorative effect of *Mfge8*‐OE on ATP content and antioxidant capacity in the in vitro AP model (*p *< .05). Consistently, the *Mfge8*‐OE‐induced reduction in intracellular Fe^2+^ levels in in vitro AP was reversed by *Abce1*‐KO, leading to increased Fe^2+^ accumulation (Figure 4E). Similar trends were observed for mitochondrial function (Figure 4F‒H), mitophagy flux (Figure 4I,J), lipid peroxidation levels (Figure 4K) and ferroptosis incidence (Figure 4L,M) in the in vitro AP model. Collectively, these results demonstrate that MFG‐E8‐mediated restoration of mitophagy homeostasis and alleviation of ferroptosis during AP are dependent on the stable expression of ABCE1. Measurement of LDH and amylase levels in cell supernatants revealed that *Abce1*‐KO antagonised the protective effect of *Mfge8*‐OE against acinar cell injury in the in vitro AP model (*p *< .05, Figure 4N,O).

### 3.5 Promoting mitophagy recovery attenuates the aggravation of in vitro AP caused by dysregulation of the MFG‐E8/ABCE1 axis

We hypothesised that acinar cell‐derived MFG‐E8 promotes mitophagy function recovery through binding to ABCE1, thereby alleviating cerulein + LPS‐induced AP. However, it remains unclear whether *Mfge8*‐OE protects impaired mitochondrial function through alternative pathways, or whether *Abce1*‐KO antagonises other established protective effects of MFG‐E8 (e.g., its well‐characterised anti‐inflammatory and anti‐apoptotic activities). To delineate the specific role of mitophagy in the MFG‐E8/ABCE1 axis‐mediated regulation of mitochondrial function and acinar cell protection during AP, we employed MA‐5—a mitophagy‐restoring agent—to examine whether mitophagy restoration could rescue the functional defects caused by ABCE1 deficiency.

**FIGURE 5:**
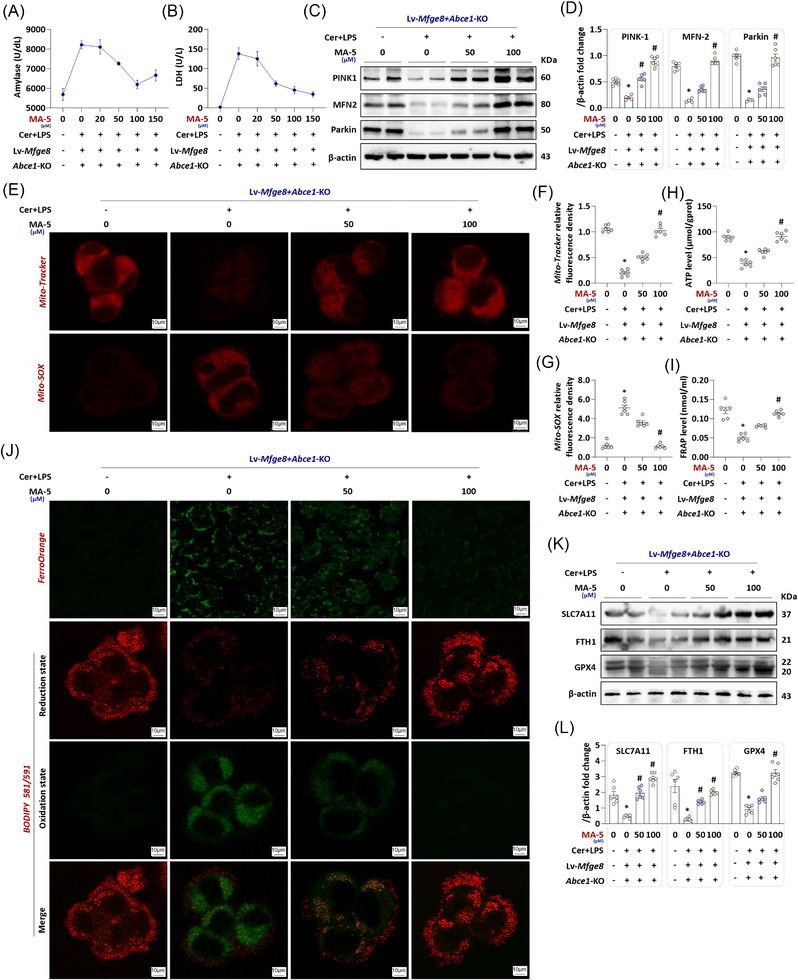
Promoting the recovery of mitophagy alleviates the aggravation of acute pancreatitis (AP) in vitro caused by the milk fat globule—epidermal growth factor 8 (MFG‐E8)/adenosine triphosphate (ATP)‐binding cassette subfamily E member 1 (ABCE1) axis disorder. (A) Amylase levels in supernatant. (B) Lactate dehydrogenase (LDH) levels in supernatant. (C and D) Western blot analysis of the PINK1, Mitofusin 2 (MFN2) and Parkin expression level in AR42J cells. (E) Representative images of Mito‐SOX and Mito‐Tracker red (1500×) in AR42J cells. (F and G) Relative fluorescence intensity of Mito‐SOX and Mito‐Tracker red in AR42J cells. (H) ATP levels in AR42J cells. (I) Ferric reducing antioxidant power (FRAP) levels in AR42J cells. (J) Representative images of FerroOrange and BODIPY 581/591 (1500×) in AR42J cells. (K and L) Western blot analysis of the solute carrier family 7 member 11 (SLC7A11), ferritin heavy chain 1 (FTH1) and glutathione peroxidase (GPX4) expression level in AR42J cells. *n* = 3‒6, error bars indicate the SEM; ^*^ *p* < .05 versus vehicle (Lv‐*Mfge8 *+ *Abce1*‐KO); ^#^ *p* < .05 versus vehicle‐AP. Cer, cerulein; FRAP, ferric reducing antioxidant power; LDH, lactate dehydrogenase; LPS, lipopolysaccharide; Lv, lentivirus; MA‐5, mitochonic acid 5; KO, knockout.

As shown in the results of LDH and amylase in Figure 5A,B, with the increase of MA‐5 concentration, the aggravation of AP in vitro caused by *Mfge8*‐OE + *Abce1*‐KO gradually alleviated. The levels of LDH and amylase in the supernatant decreased significantly when the concentration of MA‐5 was 50 µM, while they dropped to the lowest point when the concentration of MA‐5 reached 100 µM, and were basically the same as those in the control group (*p *< .05). Based on these findings, we selected the two most responsive MA‐5 concentrations for further analyses. As shown in Figure 5C,D, the mitophagy proteins PINK1, Mfn2 and Parkin were progressively rebounded with the increase of MA‐5 concentration. Similarly, MA‐5 exerted a dose‐dependent effect on mitochondrial quantity, activity and antioxidant capacity recovery (Figure 5E‒I). Furthermore, MA‐5 application attenuated intracellular Fe^2+^ accumulation and lipid peroxidation in AR42J cells (Figure 5J), concomitant with restored expression of ferroptosis‐suppressive proteins (Figure 5K,L).

### 3.6 Inhibiting ferroptosis attenuates the exacerbation of in vitro AP induced by MFG‐E8/ABCE1 axis dysregulation

**FIGURE 6:**
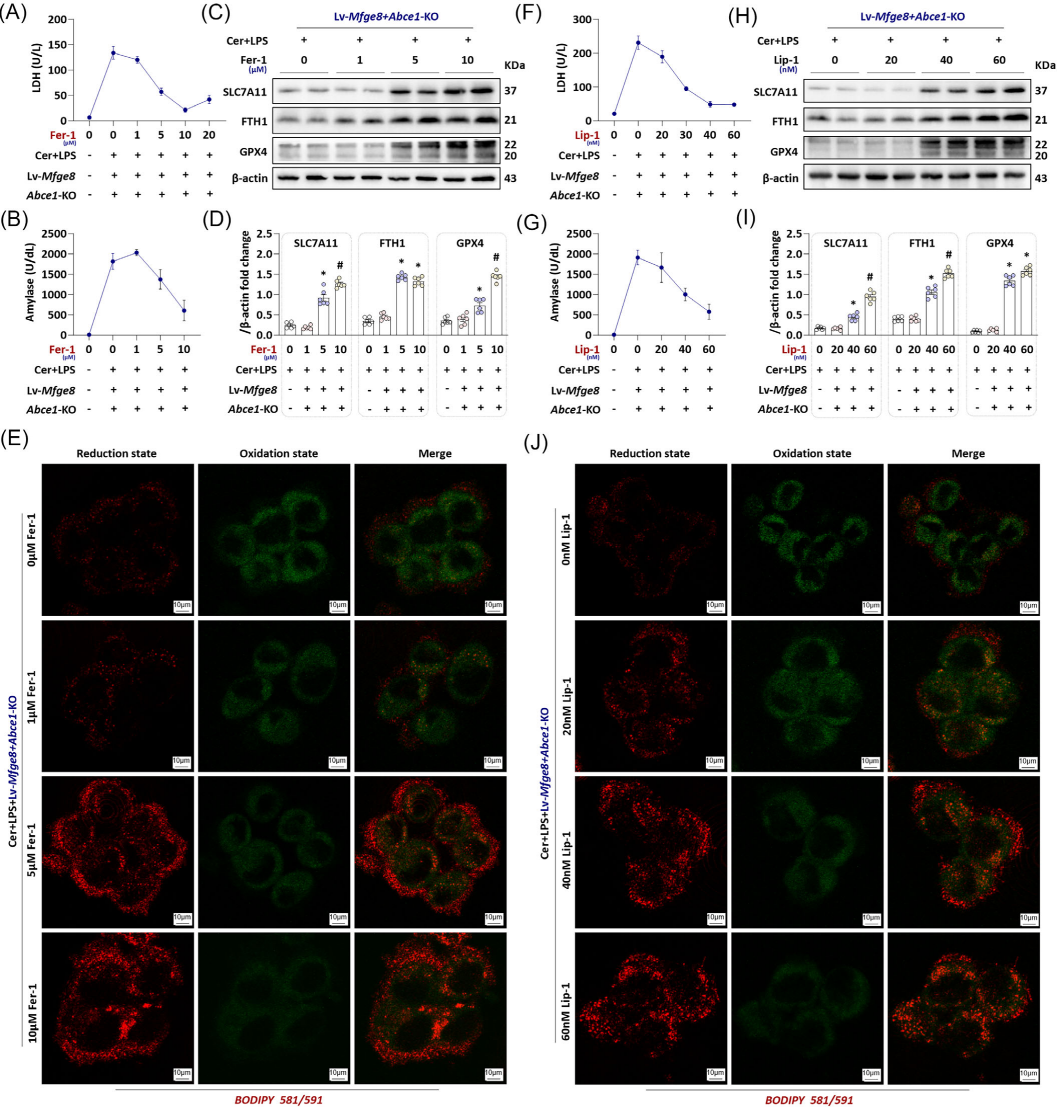
Inhibiting ferroptosis alleviates the aggravation of acute pancreatitis (AP) in vitro caused by the milk fat globule—epidermal growth factor 8 (MFG‐E8)/adenosine triphosphate (ATP)‐binding cassette subfamily E member 1 (ABCE1) axis disorder. (A) Supernatant lactate dehydrogenase (LDH) levels. (B) Supernatant amylase levels. (C and D) Western blot analysis of the solute carrier family 7 member 11 (SLC7A11), ferritin heavy chain 1 (FTH1) and glutathione peroxidase (GPX4) expression level in AR42J cells. (E) Representative images of BODIPY 581/591 (1500×) in AR42J cells. (F) Supernatant LDH levels. (G) Supernatant amylase levels. (H and I) Western blot analysis of the SLC7A11, FTH1 and GPX4 expression level in AR42J cells. (J) Representative images of BODIPY 581/591 (1500×) in AR42J cells. *n* = 3‒6, error bars indicate the SEM; ^*^ *p* < .05 versus 0 µM Ferrostatin‐1 (Fer‐1) or 0 nM Liproxstatin‐1 (Lip‐1); ^#^ *p* < .05 versus 5 µM Fer‐1 or 40 nM Lip‐1. Cer, cerulein; KO, knockout; LPS, lipopolysaccharide; Lv, lentivirus.

Oxidative/antioxidative imbalance induced by mitophagy dysfunction is a key driver of lipid peroxidation and subsequent cellular ferroptosis. We therefore hypothesised that the MFG‐E8/ABCE1 interaction inhibits ferroptosis by regulating mitophagy, ultimately reducing acinar cell death during AP progression. To confirm the critical role of ferroptosis in the MFG‐E8/ABCE1 axis‐mediated protective mechanism, we employed two distinct ferroptosis inhibitors (both in vitro and in vivo) and re‐established the AP model to assess rescue effects. As shown in Figure 6A, treatment with the ferroptosis inhibitor Fer‐1 dose dependently alleviated the exacerbation of in vitro AP caused by MFG‐E8/ABCE1 axis dysregulation. The protective effect of Fer‐1 on acinar cells was most pronounced at a concentration of 10 µM (*p *< .05). Consistent trends were observed for amylase levels in cell supernatants (Figure 6B) and the expression of ferroptosis‐related proteins (Figure 6C,D). BODIPY 581/591 fluorescence staining further confirmed Fer‐1‐inhibitory effect on ferroptosis, as evidenced by a progressive shift in lipid status from oxidised (green fluorescence) to reduced (red fluorescence) (Figure 6E). To validate these findings, we tested a second ferroptosis inhibitor, Lip‐1. As shown in Figure 6F‒J, Lip‐1 effectively mitigated lipid peroxidation and ferroptosis in the in vitro AP model at concentrations ranging from 40 to 60 µM, with a concomitant reduction in acinar cell death.

### 3.7 Ferroptosis inhibition counteracts the aggravating impact of *Mfge8*‐KO on experimental AP

**FIGURE 7:**
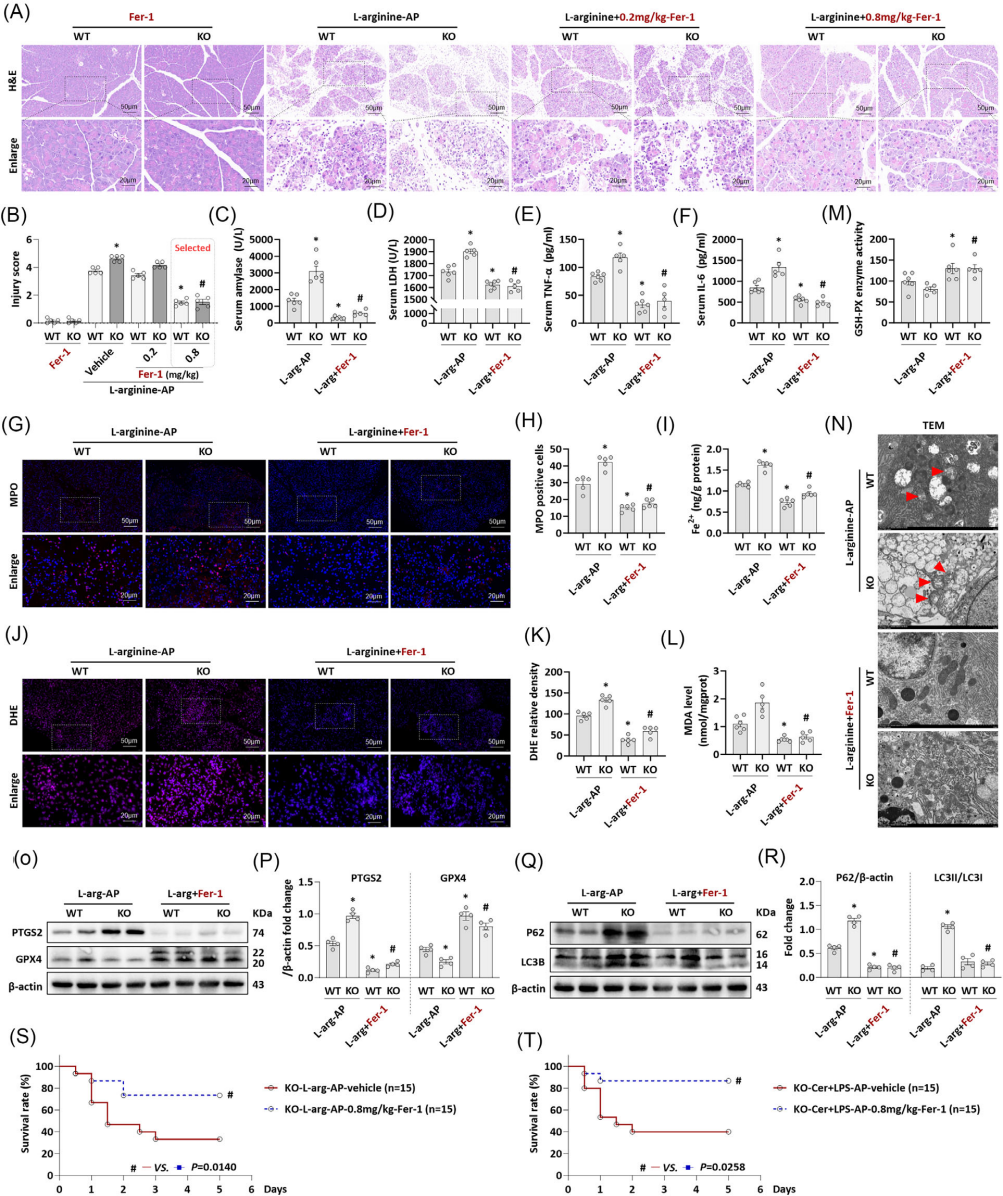
Inhibition of ferroptosis antagonised the aggravating effect of milk fat globule—epidermal growth factor 8 knockout (MFG‐E8‐KO) on experimental acute pancreatitis (AP). (A) Representative images of haematoxylin and eosin (H&E) staining of the pancreas (200×). (B) Pancreatic injury scores. (C) Serum amylase levels. (D) Serum lactate dehydrogenase (LDH) levels. (E) Serum tumour necrosis factor‐alpha (TNF‐α) levels. (F) Serum interleukin‐6 (IL‐6) levels. (G) Representative images of Myeloperoxidase (MPO) staining. (H) Quantitative of MPO staining. (I) Iron levels in the pancreas. (J and K) Representative images and relative fluorescence intensity of dihydroethidium (DHE) staining in the pancreas. (L) Malondialdehyde (MDA) levels in the pancreas. (M) GSH‐Px enzyme activity. (N) Ultrastructural alterations in the pancreas. (O and P) Western blot analysis of the PTGS2 and glutathione peroxidase (GPX4) expression level in the pancreas. (Q and R) Western blot analysis of the P62 and LC3B expression level in the pancreas. (S) Five‐day survival of L‐arginine‐AP mice. (T) Five‐day survival of cerulein + LPS‐AP mice. *n* = 4‒15, error bars indicate the SEM; ^*^ *p* < .05 versus WT‐AP; ^#^ *p* < .05 versus KO‐AP. Fer‐1, Ferrostatin‐1; L‐arg, L‐arginine; TEM, transmission electron microscopy; WT, wild type.

As previously demonstrated, endogenous MFG‐E8 deficiency (*Mfge8*‐KO) exacerbates ferroptosis in pancreatic cells of AP mice (Figure 1). However, the critical role of ferroptosis in the exacerbation of AP induced by MFG‐E8 deficiency remains to be further elucidated. As shown in Figures 7A,B and S3A,B, *mfge8*‐KO exacerbated the pancreatic tissue damage of L‐arginine‐AP mice. Injection of Fer‐1 dose dependently alleviated pancreatic injury in both WT and *Mfge8*‐KO mice: pathological improvements reached statistical significance at a Fer‐1 dose of  .8 mg/kg (*p* < .05). Notably, further increasing the Fer‐1 dose to 1 mg/kg did not elicit additional improvements in pancreatic pathology compared to the  .8 mg/kg group. These results confirm that  .8 mg/kg Fer‐1 effectively mitigates the exacerbation of AP‐associated pancreatic injury caused by *Mfge8* deficiency, likely via ferroptosis inhibition.

We therefore selected  .8 mg/kg Fer‐1 for subsequent in vivo experiments. As illustrated in Figure 7C–H, ferroptosis inhibition by Fer‐1 reversed multiple pathological hallmarks of AP exacerbated by *Mfge8*‐KO, including elevated serum amylase (Figure 7C) and LDH levels (Figure 7D), increased production of inflammatory mediators (Figure 7E,F), and enhanced inflammatory cell infiltration in pancreatic tissues (Figure 7G,H). Furthermore,  .8 mg/kg Fer‐1 normalised Fe^2+^ metabolism (Figure 7I), reduced oxidative stress (Figure 7J,K), ameliorated lipid metabolic dysfunction (Figure 7L,M) and suppressed ferroptosis (Figure 7N–P) in the pancreas of *Mfge8*‐KO AP mice. Concomitantly, Fer‐1 restored the impairment of autophagy flux (Figure 7Q,R). However,  .8 mg/kg‐Fer‐1 treatment failed to reverse the reduction of MFG‐E8's level in this L‐arginine‐induced AP models (Figure S4, *p* > .05). In *mfge8*‐KO mice, MFG‐E8 remained undetectable with or without Fer‐1, confirming no off‐target induction. This supports a hierarchical mechanism: MFG‐E8 acts upstream to suppress ferroptosis via the MFG‐E8/ABCE1‐mitophagy axis, while Fer‐1 targets downstream lipid peroxidation.

To further assess the function of Fer‐1 in MFG‐E8‐deficient AP mice, a 5‐day survival assay was performed. As shown in Figure 7S, of the 15 *Mfge8*‐KO mice with L‐arginine‐AP, five succumbed within the first day, with one death occurring within 12 h post‐AP induction. And an additional five succumbed over the following 1‒2 days. By the end of the experiment (day 5), the cumulative mortality reached 10 out of 15 mice, resulting in a survival rate of 33%. In contrast, administration of  .8 mg/kg Fer‐1 significantly improved survival: only four out of 15 mice died within the first 2 days, yielding a final survival rate of 73% (*p* < .05). Similarly, in the cerulein + LPS‐induced AP model (also *Mfge8*‐deficient), treatment with  .8 mg/kg Fer‐1 increased the survival rate from approximately 40% to over 80% (*p* < .05, Figure 7T).

### 3.8 Serum MFG‐E8 levels correlate negatively with AP severity, and exogenous MFG‐E8 restores mitophagy and attenuates ferroptosis in experimental AP

**FIGURE 8:**
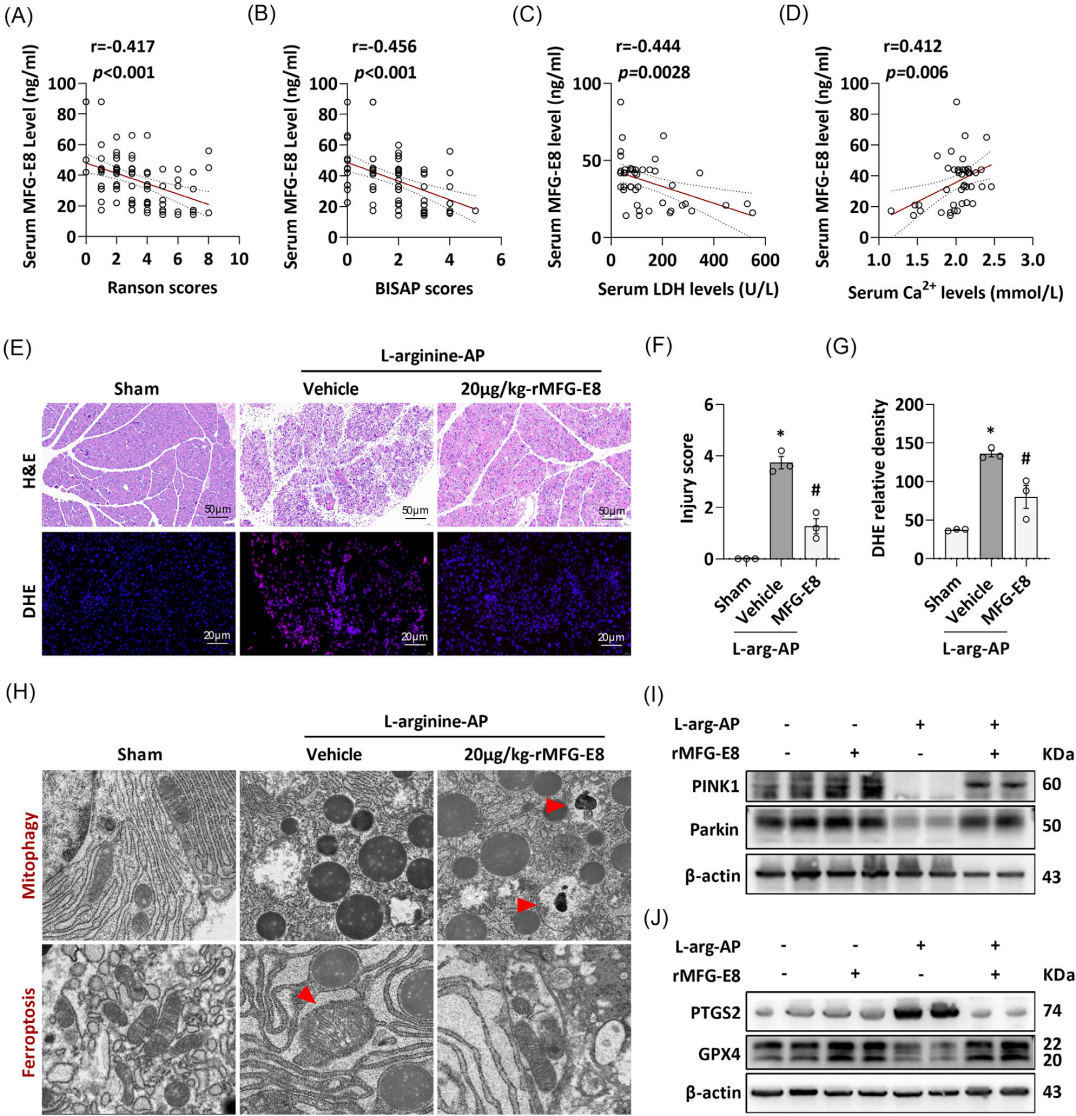
Serum milk fat globule—epidermal growth factor 8 (MFG‐E8) levels were negatively correlated with the severity of acute pancreatitis (AP), and exogenous MFG‐E8 administration restored mitophagy and attenuated ferroptosis in experimental AP. (A) Correlation analysis of serum MFG‐E8 levels and RANSON scores (*n* = 85). (B) Correlation analysis of serum MFG‐E8 levels and BISAP scores (*n* = 85). (C) Correlation analysis of serum MFG‐E8 levels and serum lactate dehydrogenase (LDH) levels (*n* = 43). (D) Correlation analysis of serum MFG‐E8 levels and serum Ca^2+^ levels (*n* = 43). (E) Representative images of haematoxylin and eosin (H&E) staining and dihydroethidium (DHE) staining of the pancreas (200×). (F) Pancreatic injury scores (*n* = 3). (G) Relative fluorescence intensity of DHE staining in the pancreas (*n* = 3). (H) Ultrastructural alterations in the pancreas. (I) Western blot analysis of the PINK1 and Parkin expression level in the pancreas. (J) Western blot analysis of the PTGS2 and glutathione peroxidase (GPX4) expression level in the pancreas.

By analysing the correlation between the serum expression level of MFG‐E8 in 85 AP patients and indicators related to the severity of this disease, we came to a conclusion similar to that of previous studies (patients’ characteristics are presented in Table S1).^10^ As shown in Figure 8A,B, serum MFG‐E8 concentrations were negatively correlated with the Ranson score (*r* = ‒.417, *p* < .01) and BISAP score (*r* = ‒.456, *p* < .01) in those AP patients, and the higher the RANSON or BISAP score, the more severe the patient's condition. The analysis of LDH levels in the serum also confirmed this conclusion (*r* = ‒.444, *p* < .01, Figure 8C). Hypocalcaemia indicates aggravated pancreatic damage, and Figure 8D shows that the serum level of MFGE8 in AP patients decreases with the decline of serum calcium (*r* = ‒.412, *p* < .01). To further elucidate the mechanism underlying the pancreatic protective effect of exogenous MFG‐E8, we re‐induced the L‐arginine‐AP model. As shown in Figure 8E‒G, 20 µg/kg of MFG‐E8, similar to previous studies, effectively alleviated pancreatic damage and oxidative stress in AP mice. More crucially, this dose of exogenous MFG‐E8 markedly restored impaired mitophagy and attenuated ferroptosis in AP mice (Figure 8H). Furthermore, exogenous MFG‐E8 restored the expression of both the PINK1‒Parkin pathway and ferroptosis‐related proteins simultaneously (Figure 8I,J).

## 4 DISCUSSION

Mitophagy selectively eliminates damaged mitochondria, preventing excessive ROS and the release of pro‐apoptotic factors, thereby maintaining the stability of the intracellular environment. In the AP model, stimuli such as increased pancreatic duct pressure and abnormal activation of pancreatic enzymes directly damage mitochondria, leading to a decrease in mitochondrial membrane potential and a significant accumulation of ROS.^22^ Under normal circumstances, the mitophagy pathway (such as the Parkin‒PINK1 pathway) is activated through ubiquitination marking damaged mitochondria and fusing with autophagosomes for degradation.^23^ However, studies have shown that in the AP state, there are abnormal dual regulatory dysregulations in mitophagy: on one hand, over‐activated inflammatory signals (such as the NF‐κB and MAPK pathways) inhibit the recruitment of Parkin, hindering the initiation of mitophagy; on the other hand, dysfunction of the autophagolysosomal system (such as the disruption of the acidic environment of lysosomes) leads to the obstruction of the clearance of damaged mitochondria, resulting in ‘mitophagy stress’.^24^, ^25^ This disorder not only exacerbates mitochondrial dysfunction but also triggers the apoptotic pathway by releasing substances such as cytochrome c, while the continuous accumulation of ROS creates conditions for ferroptosis.^26^, ^27^

Mfn2 serves as a key molecule in regulating mitochondrial dynamics. Its role in maintaining mitochondrial morphology, function and cellular homeostasis has been widely confirmed.^28^, ^29^, ^30^ Recent studies have revealed that Mfn2 plays a dual regulatory role in mitophagy. Under normal physiological conditions, Mfn2 acts as a ‘molecular bridge’ for mitophagy. By directly interacting with autophagy‐related proteins (such as LC3, p62/SQSTM1), it targets damaged mitochondria to autophagosomes, thereby initiating the selective degradation process.^31^ Additionally, Mfn2 can also affect Ca^2+^ homeostasis and ROS production by regulating the formation of mitochondrial‐associated endoplasmic reticulum (ER) membrane contacts, thereby indirectly regulating the sensitivity of mitophagy.^32^ However, under pathological conditions, abnormal expression or activity of Mfn2 may lead to dysfunction of mitophagy.^33^ For instance, in the Parkinson's disease model, the mutation of Mfn2 can inhibit the Parkin‐mediated mitochondrial ubiquitination, hinder the docking of autophagosomes with mitochondria, leading to the accumulation of damaged mitochondria and the death of dopaminergic neurons.^32^ In the AP model, we also observed changes in the expression levels of Mfn2, and its expression level recovered along with the restoration of mitophagy, indicating that Mfn2 may be involved in the regulation of mitophagy function homeostasis in AP.

The mechanism of ferroptosis in AP involves three core aspects: iron metabolism imbalance, uncontrolled lipid peroxidation and depletion of the antioxidant system.^34^ Pancreatic acinar cells (PACs) are rich in unsaturated fatty acids, and during AP, iron ions flow in large quantities into the PACs through transferrin receptor (TfR1), along with ROS produced by NADPH oxidase.^35^, ^36^ These factors jointly promote the accumulation of lipid hydroperoxides. GPX4, as a key molecule that inhibits ferroptosis, has its activity dependent on the supply of GSH, and the intracellular GSH level in cells is significantly reduced due to intensified oxidative stress during AP, leading to the inactivation of GPX4 and ultimately triggering the ferroptosis cascade reaction.^37^ It is worth noting that mitophagy disorder and ferroptosis are not independent events in AP, but rather they have a cascading amplification effect.^38^ The impairment of mitophagy function leads to a blocked clearance of damaged mitochondria, which can enhance the release of iron ions within the cell through ‘mitochondria‒ER interaction’, and promote the generation of lipid peroxidation substrates, providing a material basis for ferroptosis.^39^, ^40^ Conversely, the large amount of ROS produced during ferroptosis can further damage mitochondrial function and inhibit the activation of the mitophagy pathway, forming a vicious cycle. In addition, recent studies have found that key molecules regulating mitophagy (such as BNIP3, LC3) are also involved in the regulation of ferroptosis, suggesting that the two may share a core regulatory network, which provides potential targets for developing targeted intervention strategies.^41^, ^42^

Necroptosis, mediated by the RIPK1/RIPK3/MLKL cascade, differs fundamentally from ferroptosis, which is featured by iron‐dependent lipid peroxidation, GSH depletion and GPX4 inactivation.^43^ Our study verified that ferroptosis hallmarks are closely associated with MFG‐E8/ABCE1 dysregulation. Ferroptosis‐specific inhibitors and the mitophagy agonist MA‐5 markedly alleviated pancreatic injury, lipid peroxidation and Fe^2+^ accumulation in *Mfge8*‐KO mice and relevant cell models, while Fer‐1 normalised elevated inflammatory cytokines in *Mfge8*‐KO AP mice, suggesting inflammation is a secondary outcome of ferroptosis. Despite no direct detection of necroptosis markers, the specific pathophysiological features of ferroptosis in AP, intervention specificity and lack of necroptosis activation evidence confirm that phenotypic changes are mainly mediated by MFG‐E8/ABCE1‐regulated ferroptosis.

Through in vivo or in vitro study, we found that the expression level of endogenous MFG‐E8 in the AP model was significantly downregulated, and it was positively correlated with the mitophagy activity of acinar cells. This suggests that MFG‐E8 may be involved in the regulation process of mitophagy. At the mechanism level, Co‐IP indicated that MFG‐E8 could directly bind to ABCE1, a key mitophagy regulatory protein, promoting its interaction with the key mitophagy molecule PINK1, thereby activating Parkin‐mediated mitochondrial ubiquitination modification and accelerating the recognition and encapsulation of damaged mitochondria by autophagosomes. At the same time, the MFG‐E8/ABCE1 complex could regulate the lipidation process of LC3‐II, promoting the fusion of autophagosomes and lysosomes, and enhancing the degradation efficiency of mitochondria. The elucidation of this regulatory axis not only expands the biological function of ABCE1, but also provides a new molecular explanation for the role of MFG‐E8 in cellular stress responses. It is worth noting that when ABCE1 was knocked down, the promoting effect of MFG‐E8 on mitophagy and the protective effect on acinar cells were significantly weakened, confirming that ABCE1 is the key downstream molecule for the function of MFG‐E8.

The results of this study have certain correlations and extensions to previous research. Our recent studies have shown that exogenous MFG‐E8 regulates cell survival signals through integrin receptors.^10^, ^44^ However, the non‐receptor‐dependent protein‐protein interaction mechanism discovered in this study reveals a new pathway in the regulation of intracellular homeostasis. In addition, the role of ABCE1 in energy metabolism and protein synthesis suggests that it may act as a ‘sensor’ in the cellular stress state.^45^ The binding of MFG‐E8 may enhance its ability to perceive and respond to mitochondrial dysfunction. In the context of AP pathology, abnormal activation of pancreatic enzymes leading to oxidative stress and Ca^2+^ overload can induce loss of mitochondrial membrane potential and release of ROS. At this stage, MFG‐E8/ABCE1‐mediated activation of mitophagy facilitates the prompt clearance of damaged mitochondria and reduces the release of pro‐inflammatory factors as well as the incidence of cell necrosis. Consequently, this process contributes to the mitigation of local pancreatic inflammation and systemic complications. Our findings also extending MFG‐E8's role in tissue homeostasis. This conserved pathway may apply to other degenerative conditions (e.g., IVD degeneration). Translational strategies (injectable hydrogels, MFG‐E8 mimetic peptides targeting ABCE1) can improve bioavailability/specificity for AP therapy. Future studies should validate the axis in other tissues and optimise delivery systems to advance related therapeutic strategies.^46^

Exogenous and endogenous MFG‐E8 exert complementary protective effects in AP via distinct but synergistic pathways, unifying our current and previous findings. Exogenous MFG‐E8 binds integrin αVβ3/5 receptors to activate the FAK‒STAT3 pathway, primarily suppressing systemic/local inflammation and ER stress—creating a protective microenvironment that minimises secondary mitochondrial damage. In contrast, endogenous MFG‐E8 acts cell—intrinsically by directly binding ABCE1, activating Parkin‒PINK1‐mediated mitophagy to clear damaged mitochondria, reduce Fe^2+^ accumulation and lipid peroxidation, and suppress ferroptosis. Their synergism is reinforced by mutual reinforcement: mitophagy restoration via the MFG‐E8/ABCE1 axis reduces ROS production, enhancing FAK‒STAT3's anti‐inflammatory efficacy; conversely, FAK‒STAT3‐mediated inflammation suppression minimises mitochondrial insult, facilitating mitophagy. This multi‐layered mechanism allows MFG‐E8 to target AP pathogenesis comprehensively and highlighting the therapeutic potential of co‐targeting both pathways for enhanced AP treatment.

From the perspective of clinical translation, the findings of this study have potential application value. On one hand, the expression level of endogenous MFG‐E8 may serve as a biomarker for evaluating the severity of AP. Its low expression may indicate a decreased mitophagy function and an increased risk of disease progression. On the other hand, regulating the MFG‐E8/ABCE1 pathway to enhance mitophagy activity may become a new strategy for the treatment of AP. For example, mimetic peptides or small molecule agonists designed based on the interaction interface of MFG‐E8 and ABCE1 are expected to specifically activate mitophagy while avoiding interference with other autophagy processes.

However, this study still has certain limitations. First, although in vitro experiments have confirmed the direct binding of MFG‐E8 to ABCE1, in the complex microenvironment of the body, whether other proteins are involved in the formation or regulation of this complex still needs further exploration. Second, this study mainly focuses on the Parkin‐dependent mitophagy pathway, whether MFG‐E8/ABCE1 participates in other types of mitophagy regulation (such as the Nix/BNIP3‐mediated pathway) remains to be studied. Moreover, although current studies have initially revealed the synergistic effect of mitophagy disorder and ferroptosis in AP, there are still many unresolved issues. For instance, the dynamic changes in mitophagy and ferroptosis in different severity levels of AP and their correlations with clinical prognosis; the molecular mechanisms of specifically regulating the cross‐talk pathway of mitophagy‐ferroptosis; as well as the safety and efficacy of targeted intervention strategies in the process of translating animal models to clinical applications. In‐depth exploration of these scientific issues is expected to provide new theoretical basis and therapeutic targets for the precise prevention and treatment of AP.

Moreover, the use of whole‐body *mfge8*‐KO mice may introduce complexity to interpreting pancreatic acinar cell‐specific effects, as MFG‐E8 deficiency in immune and other cell types may indirectly influence pancreatic injury through systemic inflammatory responses. However, in our in vitro experiment, MFG‐E8 overexpression alone restored mitophagy flux, suppressed ferroptosis, and improved mitochondrial function independent of other cell types; Co‐IP experiments validated the direct interaction between MFG‐E8 and ABCE1 in these cells, with ABCE1 knockdown abolishing MFG‐E8's protective effects. Furthermore, AP is a systemic disorder where pancreatic acinar cell injury and systemic inflammation are mutually reinforcing, and whole‐body KO mice recapitulate this physiological interplay, while targeting the MFG‐E8/ABCE1 axis clinically may exert beneficial effects on both acinar cell injury and systemic inflammation—enhancing its translational relevance.

## 5 CONCLUSION

**FIGURE 9:**
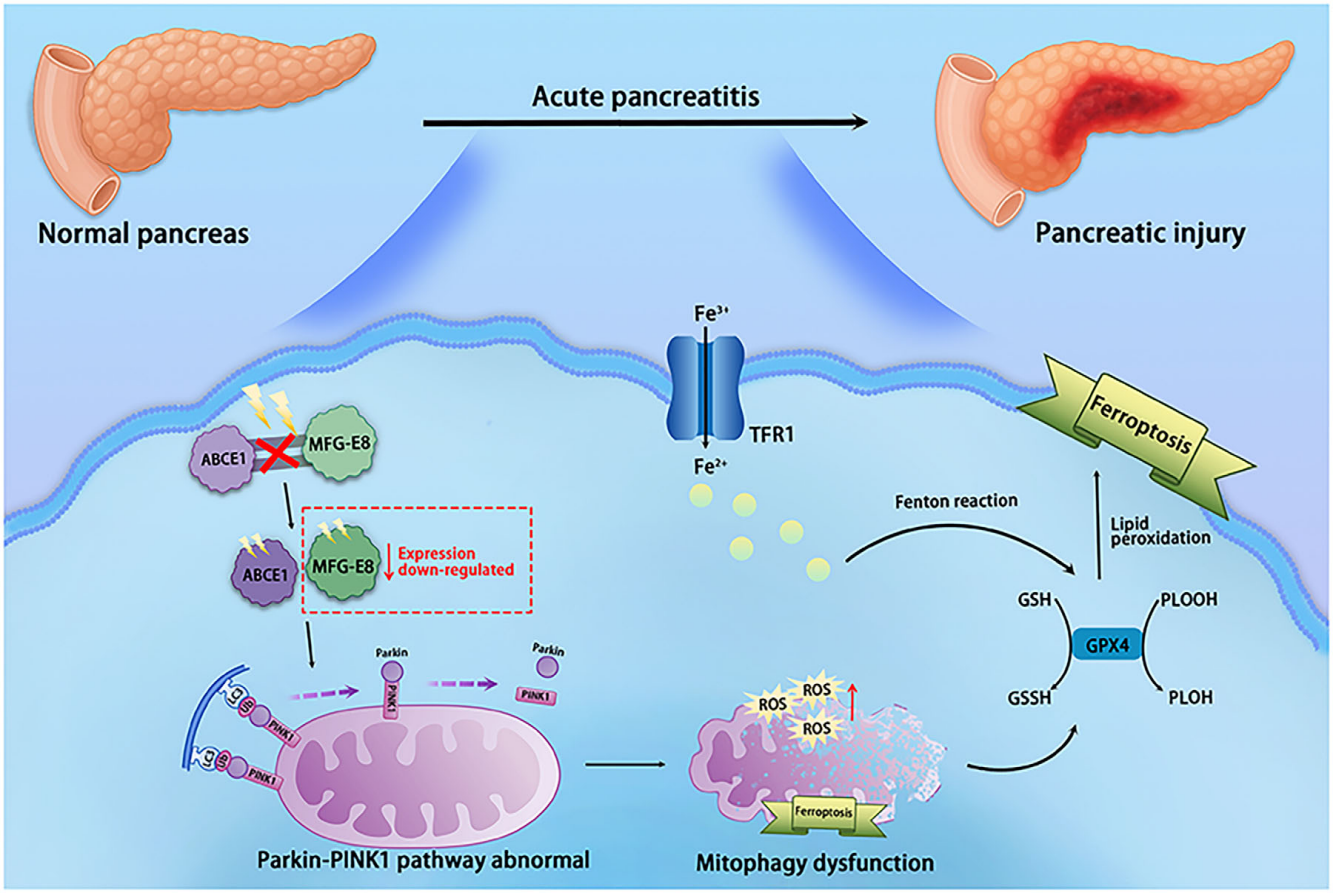
Graphical abstract. During acute pancreatitis (AP), milk fat globule—epidermal growth factor 8 (MFG‐E8) expression in pancreatic acinar cells is downregulated, resulting in disruption of the adenosine triphosphate (ATP)‐binding cassette subfamily E member 1 (ABCE1)/MFG‐E8 complex. This impairment leads to defective mitophagy flux, accumulation of lipid peroxides and ferroptosis, ultimately contributing to pancreatic damage.

MFG‐E8 specifically binds ABCE1 to form a functional complex that enhances Parkin‒PINK1‐mediated mitophagy and recruits LC3‐II to accelerate autophagosome maturation, efficiently clearing AP‐induced damaged mitochondria. This timely clearance reduces free iron release and ROS accumulation in PACs, preserves GPX4 activity to inhibit lipid peroxidation, and ultimately suppresses ferroptosis—provides a potential ‘mitochondrial quality control‐ferroptosis inhibition’ strategy for targeted AP intervention (Figure 9).

## AUTHOR CONTRIBUTIONS

Yifan Ren, Yuxuan Lu and Qing Cui acquired and analysed the data, and wrote the paper. Hao Shang, Xiali Shi, Yun Sun and Meng Fan interpreted the data. Yifan Ren and Rongqian Wu interpreted the data and revised the paper. Yifan Ren and Hongwei Lu designed and supervised the study and revised the paper. All authors have read and agreed with the final manuscript.

## CONFLICT OF INTEREST STATEMENT

The authors declare that the research was conducted in the absence of any commercial or financial relationships that could be construed as a potential conflict of interest.

## ETHICS STATEMENT

All experiments were performed in accordance with the guidelines of the China Council on Animal Care and Use and approved by the Institutional Animal Care and Use Committee of the Ethics Committee of Xi'an Jiaotong University Health Science Center.

## Supporting information



FIGURE S1. The *mfge8* gene knockout mice were created. Western blot analysis of the milk fat globule—epidermal growth factor 8 (MFG‐E8) expression level in the pancreas. *n* = 6, error bars indicate the SEM; ^*^ *p* < .05. KO, knockout; WT, wild type.

FIGURE S2. Construct an AR42J cell line with *Mfge8* overexpression and adenosine triphosphate (ATP)‐binding cassette subfamily E member 1 knockout (ABCE1‐KO) dual gene modification. Western blot analysis of the milk fat globule—epidermal growth factor 8 (MFG‐E8) and ABCE1 expression level in AR42J. *n* = 12, error bars indicate the SEM; ^*^ *p* < .05. Lv, lentivirus.

FIGURE S3. Inhibition of ferroptosis antagonised the aggravating effect of milk fat globule—epidermal growth factor 8 knockout (MFG‐E8‐KO) on experimental acute pancreatitis (AP). (A) Representative images of haematoxylin and eosin (H&E) staining of the pancreas (200×). (B) Pancreatic injury scores. *n* = 6, error bars indicate the SEM; ^*^ *p* < .05. Fer‐1, Ferrostatin‐1; LPS, lipopolysaccharide; WT, wild type.

FIGURE S4. Ferroptosis inhibition have no effect on the expression level of milk fat globule—epidermal growth factor 8 (MFG‐E8). Western blot analysis of the MFG‐E8 expression level in the pancreas. *n* = 4, error bars indicate the SEM; N.S., no significant differences. Fer‐1, Ferrostatin‐1; MFG‐E8, milk fat globule—epidermal growth factor 8; KO, knockout; WT, wild type.

TABLE S1. Characteristics of patients with acute pancreatitis.

FIGURE S5. Entire membranes of the representative Western blot in Figure 1.
FIGURE S6. Entire membranes of the representative Western blot in Figure 2.
FIGURE S7. Entire membranes of the representative Western blot in Figure 3.
FIGURE S8. Entire membranes of the representative Western blot in Figure 4.
FIGURE S9. Entire membranes of the representative Western blot in Figure 5.
FIGURE S10. Entire membranes of the representative Western blot in Figure 6.
FIGURE S11. Entire membranes of the representative Western blot in Figure 7.
FIGURE S12. Entire membranes of the representative Western blot in Figure 8.
FIGURE S13. Entire membranes of the representative Western blot in Figure S1.
FIGURE S14. Entire membranes of the representative Western blot in Figure S2.
FIGURE S15. Entire membranes of the representative Western blot in Figure S4.
TABLE S2. Antibodies.

## Data Availability

Data used to support the findings of this study are available from the corresponding author upon request.
