# Supplementary figures and images for: Milk fat globule—EGF factor 8/ATP‐binding cassette subfamily E member 1 axis maintains mitophagy flux homeostasis to suppress ferroptosis in acute pancreatitis
Source: Clin Transl Med. 2026 Feb 18;16(2):e70619. doi: 10.1002/ctm2.70619 (PMC12914346; doi:10.1002/ctm2.70619)

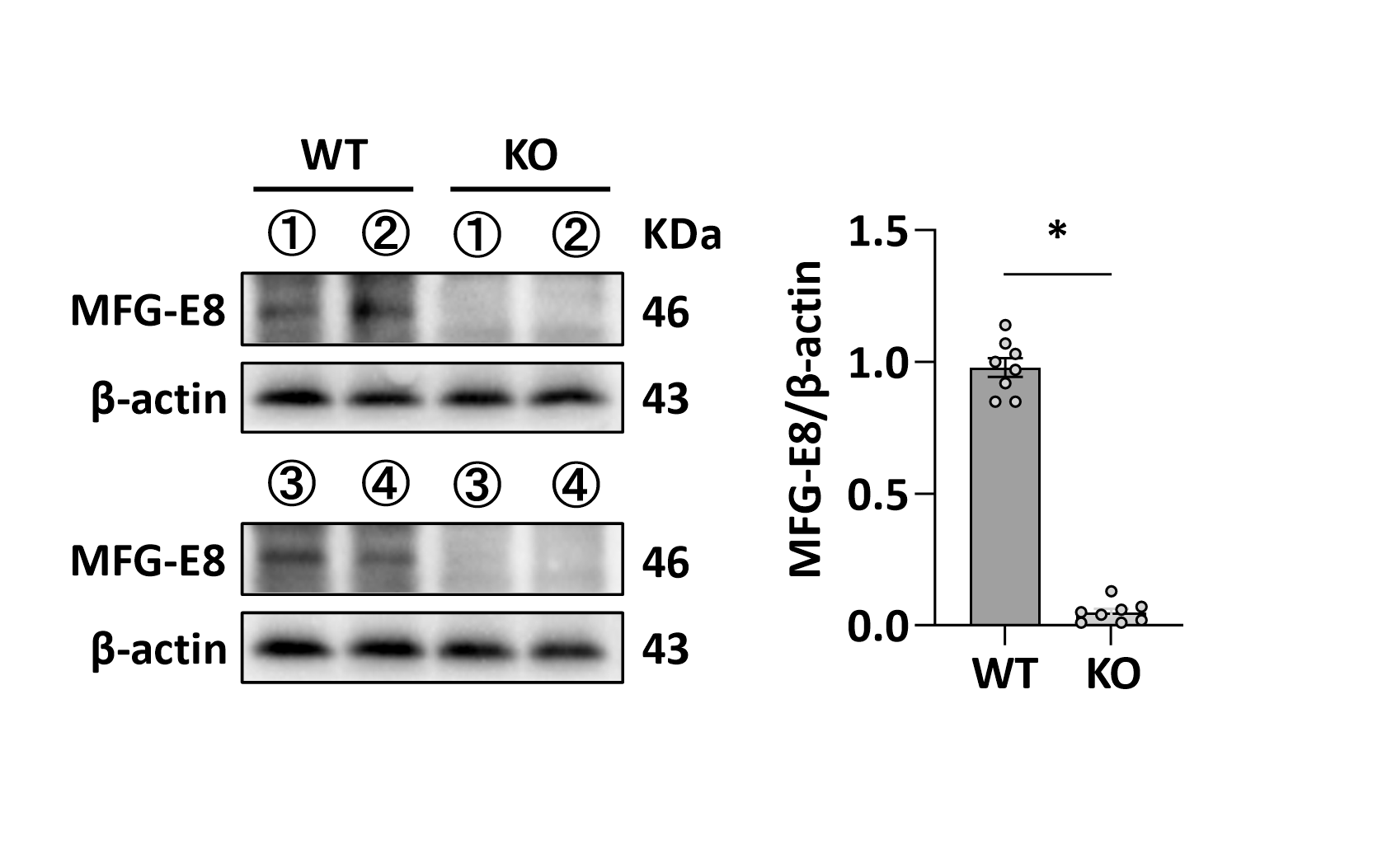

Supplement: Supplementary file 1 — FIGURE S1. The mfge8 gene knockout mice were created. Western blot analysis of the milk fat globule—epidermal growth factor 8 (MFG‐E8) expression level in the pancreas. n = 6, error bars indicate the SEM; * p < .05. KO, knockout; WT, wild type. [file CTM2-16-e70619-s006.tif]

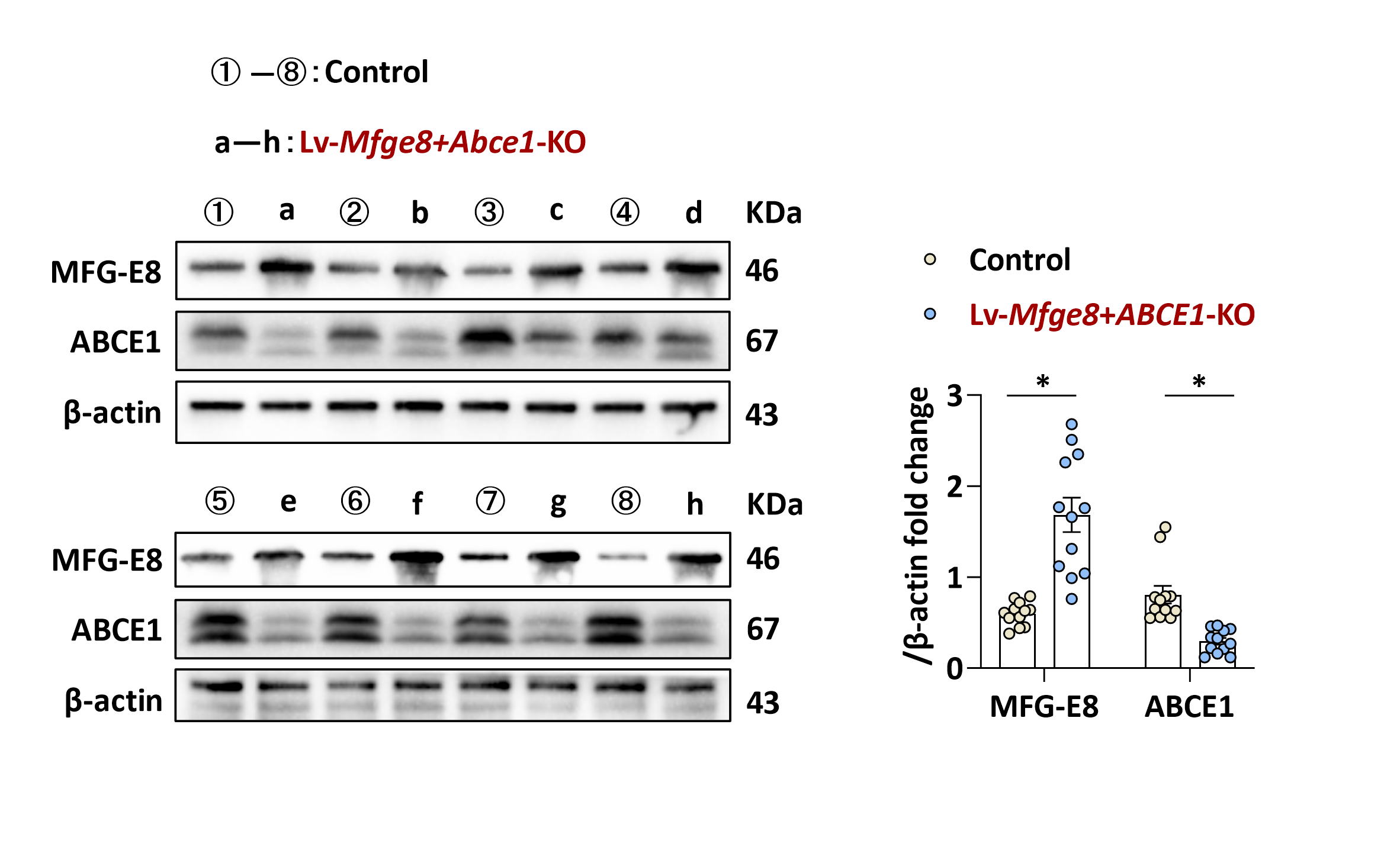

Supplement: Supplementary file 2 — FIGURE S2. Construct an AR42J cell line with Mfge8 overexpression and adenosine triphosphate (ATP)‐binding cassette subfamily E member 1 knockout (ABCE1‐KO) dual gene modification. Western blot analysis of the milk fat globule—epidermal growth factor 8 (MFG‐E8) and ABCE1 expression level in AR42J. n = 12, error bars indicate the SEM; * p < .05. Lv, lentivirus. [file CTM2-16-e70619-s001.tif]

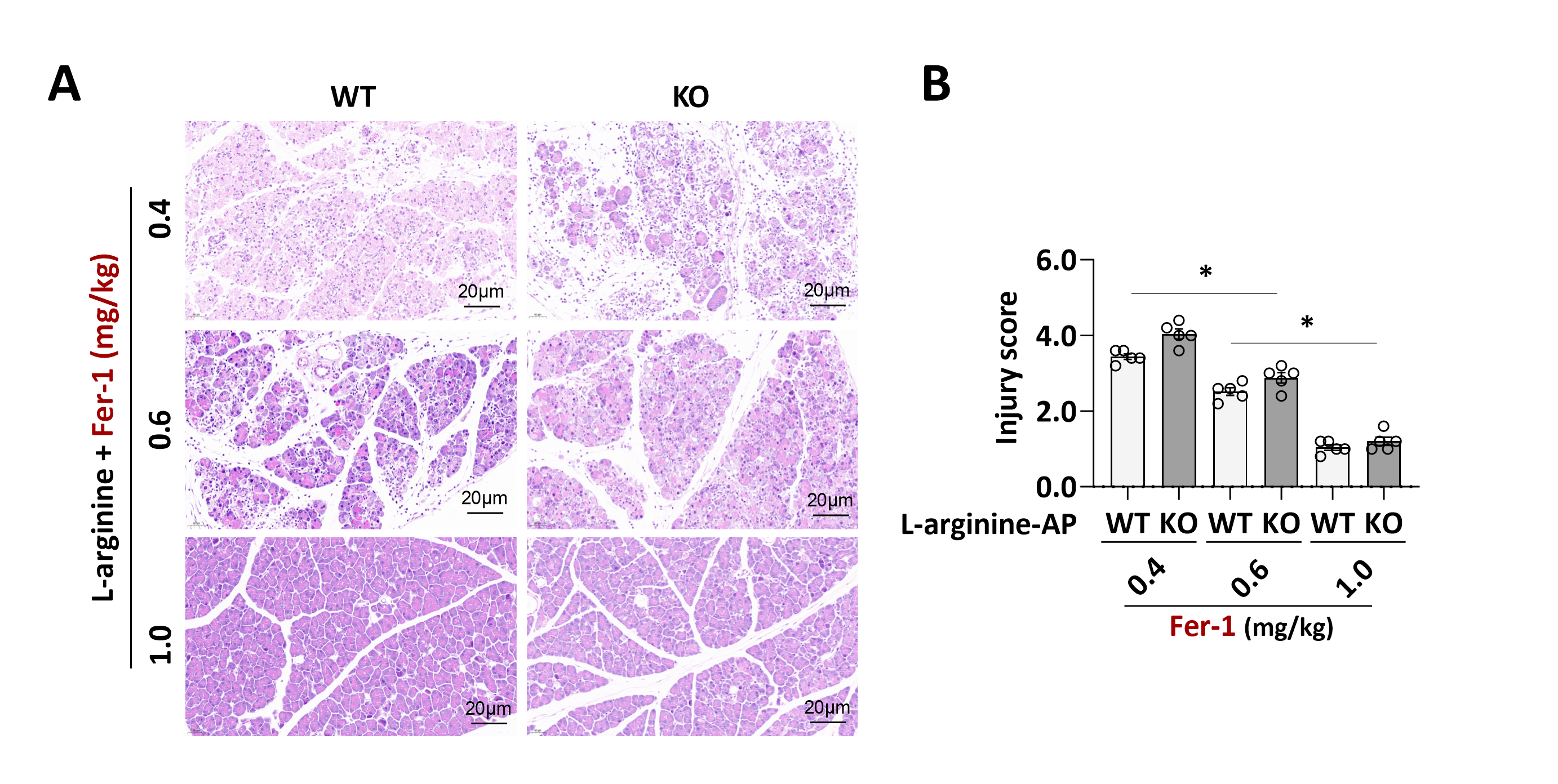

Supplement: Supplementary file 3 — FIGURE S3. Inhibition of ferroptosis antagonised the aggravating effect of milk fat globule—epidermal growth factor 8 knockout (MFG‐E8‐KO) on experimental acute pancreatitis (AP). (A) Representative images of haematoxylin and eosin (H&E) staining of the pancreas (200×). (B) Pancreatic injury scores. n = 6, error bars indicate the SEM; * p < .05. Fer‐1, Ferrostatin‐1; LPS, lipopolysaccharide; WT, wild type. [file CTM2-16-e70619-s005.tif]

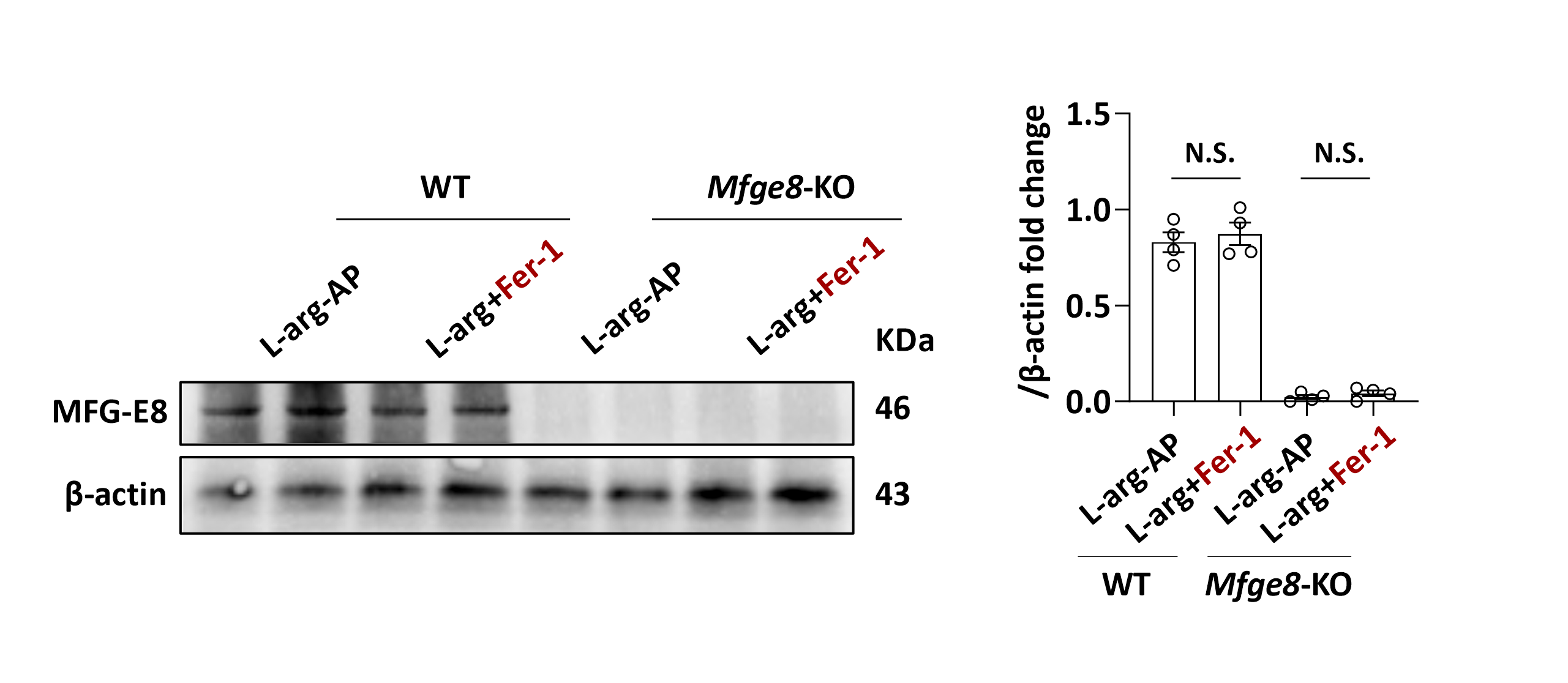

Supplement: Supplementary file 4 — FIGURE S4. Ferroptosis inhibition have no effect on the expression level of milk fat globule—epidermal growth factor 8 (MFG‐E8). Western blot analysis of the MFG‐E8 expression level in the pancreas. n = 4, error bars indicate the SEM; N.S., no significant differences. Fer‐1, Ferrostatin‐1; MFG‐E8, milk fat globule—epidermal growth factor 8; KO, knockout; WT, wild type. [file CTM2-16-e70619-s002.tif]
