# Supplementary material for: Milk fat globule—EGF factor 8/ATP‐binding cassette subfamily E member 1 axis maintains mitophagy flux homeostasis to suppress ferroptosis in acute pancreatitis
Source: Clin Transl Med. 2026 Feb 18;16(2):e70619. doi: 10.1002/ctm2.70619 (PMC12914346; doi:10.1002/ctm2.70619)
Supplement: Supplementary file 5 — TABLE S1. Characteristics of patients with acute pancreatitis. [file CTM2-16-e70619-s003.docx]

**Supplementary Table 1: Characteristics of patients with acute pancreatitis.**

| **Characteristics** | **N (%) or mean ± SD** |  |
| --- | --- | --- |
| Number | 85 |  |
| Age (Year) | 48 ± 14.3 |  |
| Sex (male/female)  Body Mass Index | 53/32  22.0 ± 1.9 |  |
| *Classification*  Mild  Moderately severe  Severe | 51 (60.0%)  16 (18.8%)  18 (21.2%) |  |
| *Etiology* |  |  |
| Biliary | 34 (40.0%) |  |
| Alcoholic | 2 (2.4%) |  |
| Hypertriglyceremic | 22 (25.9%) |  |
| Others | 27 (31.7%) |  |
| *Treatments* |  |  |
| Conservative therapy | 61 (71.8%) |  |
| Percutaneous drainage | 6 (7.1%) |  |
| Laparotomy | 18 (21.1%) |  |
| APACHE II scores | 5.8 ± 5 |  |
| SOFA scores | 2.1 ± 3 |  |
| Local complication (Yes/No) | 21/64 |  |
| Organ failure (Yes/No) | 13/72 |  |
| Serum PCT (ng/ml) | 2.8 ± 6.4 |  |
| Serum CRP (mg/L) | 135.5 ± 166 |  |
| Serum lipase (U/L) | 1752.8 ± 1831 |  |
| Serum amylase (U/L) | 713.3 ± 963.7 |  |
| Blood Glucose(mmol/L)  HbA1c (%)  WBC(*10^9/L)  Serum creatinine(μmol/L)  Serum BUN (mmol/L)  HCT (%)  Serum calcium(mmol/L) | 8.5 ± 7.3  5.2 ± 2.1  11.6 ± 5.9  87.7 ± 99  7.3 ± 91  39 ± 11.5  2.1 ± 0.2 |  |

**Supplementary Table 2: Antibodies**

| **Antibody** | **Item No** | **Company and location** |
| --- | --- | --- |
| β-Actin Mouse Monoclonal Antibody | AF0003 | Beyotime Biotechnology, CN |
| SQSTM1/p62 Antibody | 5114 | Cell Signaling Technology, Beverly, MA, USA |
| FTH1 (D1D4) Rabbit mAb | 4393 | Cell Signaling Technology, Beverly, MA, USA |
| SLC7A11 Rabbit Polyclonal Antibody | AF7992 | Beyotime Biotechnology, CN |
| LC3B Antibody | 2775 | Cell Signaling Technology, Beverly, MA, USA |
| Cox2 (D5H5) Rabbit mAb (PTGS2) | 73315 | Cell Signaling Technology, Beverly, MA, USA |
| GPX4 (E5Y8K) Rabbit mAb | 59735 | Cell Signaling Technology, Beverly, MA, USA |
| PINK1 Rabbit Polyclonal Antibody | Af7755 | Beyotime Biotechnology, CN |
| MFG-E8 Antibody | sc-271574 | Santa Cruz Biotechnology, Inc. |
| ABCE1 Antibody | sc-518185 | Santa Cruz Biotechnology, Inc. |
| Normal Mouse IgG | A7028 | Beyotime Biotechnology, CN |
| Myeloperoxidase Rabbit mAb | 15178 | Cell Signaling Technology, Beverly, MA, USA |
| FIS1 (E3K9O) Rabbit mAb | 32525 | Cell Signaling Technology, Beverly, MA, USA |
| PGC1α Rabbit Polyclonal Antibody | AF7736 | Beyotime Biotechnology, CN |
| DRP1 (D6C7) Rabbit mAb | 8570 | Cell Signaling Technology, Beverly, MA, USA |
| TFAM Rabbit Polyclonal Antibody | AF8127 | Beyotime Biotechnology, CN |
| Goat anti-Mouse IgG antibody | 31430 | PIONEER Biotechnology, CN |
| Goat anti-Rabbit IgG antibody | 31460 | PIONEER Biotechnology, CN |
| MFN2 Rabbit Polyclonal Antibody | AF7473 | Beyotime Biotechnology, CN |
