# Supplementary material for: Milk fat globule—EGF factor 8/ATP‐binding cassette subfamily E member 1 axis maintains mitophagy flux homeostasis to suppress ferroptosis in acute pancreatitis
Source: Clin Transl Med. 2026 Feb 18;16(2):e70619. doi: 10.1002/ctm2.70619 (PMC12914346; doi:10.1002/ctm2.70619)
Supplement: Supplementary file 6 — FIGURE S5. Entire membranes of the representative Western blot in Figure 1. FIGURE S6. Entire membranes of the representative Western blot in Figure 2. FIGURE S7. Entire membranes of the representative Western blot in Figure 3. FIGURE S8. Entire membranes of the representative Western blot in Figure 4. FIGURE S9. Entire membranes of the representative Western blot in Figure 5. FIGURE S10. Entire membranes of the representative Western blot in Figure 6. FIGURE S11. Entire membranes of the representative Western blot in Figure 7. FIGURE S12. Entire membranes of the representative Western blot in Figure 8. FIGURE S13. Entire membranes of the representative Western blot in Figure S1. FIGURE S14. Entire membranes of the representative Western blot in Figure S2. FIGURE S15. Entire membranes of the representative Western blot in Figure S4. TABLE S2. Antibodies. [file CTM2-16-e70619-s004.docx]

Supplementary Materials for

**The MFG-E8/ABCE1 Axis Maintains Mitophagy Flux to Suppress Ferroptosis in Acute Pancreatitis**

Yifan Ren; Yuxuan Lu; Qing Cui; Hao Shang; Meng Fan; Yun Sun; Xiali Shi; Rongqian Wu; Hongwei Lu*

*Corresponding author: lhwdoc@mail.xjtu.edu.cn.

**This file includes:**

Supplementary Materials and Methods

Supplementary Table 1;

Supplementary Table 2;

Supplementary Figures 1-15 and Figure Legends;

Report on the Construction of Stable Cell Lines.

**Supplementary Materials and Methods**

**Pathological staining:** Pancreatic tissue samples were stained with H&E. The stained sections were digitally scanned with a biopsy scanner (Pannoramic MIDI, 3DHISTECH, Hungary). The pathology score was assessed as described before. Briefly, Acinar cell damage: This parameter was evaluated based on three key manifestations: acinar cell necrosis, vacuolization, and disruption of the acinar architectural structure. The scoring scale was set as: 0 points, indicating no detectable acinar cell damage; 1 point, representing mild damage (affecting <25% of acinar cells); 3 points, corresponding to moderate damage (involving 25%–50% of acinar cells); and 5 points, signifying severe damage (impacting >50% of acinar cells). Inflammatory cell infiltration: The extent of inflammatory cell infiltration in pancreatic tissue was assessed. The scoring criteria were defined as: 0 points, indicating no inflammatory cell infiltration; 1 point, denoting mild infiltration (characterized by scattered distribution of inflammatory cells); 3 points, representing moderate infiltration (manifested as localized aggregates of inflammatory cells in partial regions); and 5 points, indicating severe infiltration (exhibiting diffuse and extensive infiltration of inflammatory cells throughout the tissue). Interstitial edema: The degree of pancreatic interstitial edema was scored according to the following standards: 0 points, indicating no edema; 1 point, representing mild edema (with slight widening of the interstitial space); 3 points, corresponding to moderate edema (with obvious separation of tissue structures by accumulated fluid); and 5 points, signifying severe edema (with massive fluid accumulation accompanied by significant distortion of tissue morphology). All scoring procedures were independently conducted by two experienced pathologists who were kept blinded to the group assignments of the experimental samples, ensuring the objectivity of the evaluation.

**Immunofluorescent staining:** Pancreatic tissue samples were fixed with 4% paraformaldehyde and then permeabilized with 0.5% Triton X-100. A primary rabbit anti-MFG-E8 antibody (sc-271574, Santa Cruz Biotechnology, Inc., 1:200 dilution) or Myeloperoxidase (E2Z8J) Rabbit mAb (15178, Cell Signaling Technology, Beverly, MA, USA) was incubated with samples overnight at 4°C. Alexa Fluor 594-conjugated Donkey Anti-Rabbit IgG(H+L) (SA00006-8, Proteintech, China, 1:200 dilution) was incubated for 1 h at room temperature. Randomly capture three different fields of view under the fluorescence microscope, and use the Image-Pro Plus 6.0 (Media Cybernetics, USA) software for quantitative analysis of fluorescence intensity.

**ATP content determination:** AR42J homogenate was obtained. The content of ATP in AR42J was detected according to the specifications of the assay kit (S0026, Beyotime, China). Briefly, prepare a series of ATP standard solutions by diluting the stock ATP solution with ATP detection lysate to create an appropriate concentration gradient, such as 0.01, 0.03, 0.1, 0.3, 1, 3, and 10 μM. The concentration range of the standards can be adjusted in subsequent experiments based on the expected ATP levels in the samples. Next, prepare the ATP test solution: calculate the total volume needed (100 μL per sample or standard), then dilute the ATP test reagent with ATP test reagent diluent at a 1:4 ratio to obtain the ATP detection working solution for use in the assay. For measuring sample ATP content, add 100 μL of the ATP detection working solution to each test tube and incubate at room temperature for 3-5 minutes to consume any background ATP and minimize interference. Then, add 20 μL of sample or standard to the test tube, mix quickly with a micropipette, and after at least 2 seconds, measure the relative light units (RLU) using a luminometer or counts per minute (CPM) using a liquid scintillation counter. Finally, determine the ATP concentration in the samples by comparing their readings to the standard curve generated from the known ATP standards.

**Transmission Electron Microscopy (TEM):** Pancreatic tissue was sectioned by resin embedding, 60-80 nm ultra-thin sections of pancreatic samples were stained with uranyl acetate and lead citrate. Pancreatic ultrastructure, such as mitochondria and endoplasmic reticulum, was evaluated using a transmission electron microscope (HITACHI HT7700, Hitachi, Japan) by a technical microscopist.

**Detection of ferrous ion (Fe^2+^) content:** The cell homogenate was prepared as required in an ice bath, and the supernatant was taken and placed on ice for testing. The 40 mmol/L standard solution was diluted with distilled water to obtain standard solutions of different concentrations. Based on the concentration and absorbance of the standard tubes, a standard curve was established. The concentration of the sample is determined based on the standard curve.

**Detection of MDA, GSH and GSH-Px level:** For the determination of malondialdehyde (MDA), glutathione (GSH) and glutathione peroxidase (GSH-Px) levels, both tissue and cell samples were processed as follows: For tissue samples (100 mg), homogenization was performed in ice-cold 0.9% physiological saline (1:9, w/v) using a tissue homogenizer, followed by centrifugation at 3,000 g for 15 min at 4°C to collect the supernatant. For cell samples (1×10⁶ cells), cells were first harvested by centrifugation at 1,000 g for 5 min at 4°C, washed twice with ice-cold phosphate-buffered saline (PBS, pH 7.4), then resuspended in 1 mL of ice-cold 0.9% physiological saline and lysed via ultrasonic disruption on ice, followed by centrifugation at 3,000 g for 15 min at 4°C to obtain the supernatant. MDA levels were measured using the TBA method: 100 μL supernatant mixed with 200 μL TBA reagent, boiled for 15 min, cooled, centrifuged at 3,000×g for 10 min, and absorbance read at 532 nm, with concentrations calculated via 1,1,3,3-tetramethoxypropane standards (expressed as nmol/mg protein) (G4300, Servicebio, Chain). GSH levels were assayed using a commercial kit with DTNB, where 50 μL supernatant mixed with 150 μL DTNB-containing buffer was incubated at 37°C for 10 min, absorbance measured at 412 nm, and concentrations calculated via GSH standards (expressed as μmol/mg protein) (G4305, Servicebio, Chain). The activity of GSH-Px in pancreatic tissue was quantified using a commercial assay kit (A005-1, Nanjing Jiancheng Bioengineering Institute, China). One unit of enzyme activity (U) was defined as the amount of enzyme required to catalyze the oxidation of 1 μmol of reduced GSH per minute per milligram of protein at 37 °C, after accounting for non-enzymatic background reactions.

**Mito-SOX, Mito-Tracker, BODIPY 581/591, FerroOrange and DHE (Dihydroethidium) Staining:** A Mito-SOX kit (S0061S, Beyotime, China), a FerroOrange kit (F374, Dojindo, China) kit, a BODIPY 581/591 kit (S0043S, Beyotime, China) kit, a MitoTracker staining kit (Mitochondrial probe, M7512, Thermo Fisher Scientific, Beijing, China) and a DHE (Dihydroethidium, G1045, Servicebio, Wuhan, China) kit were used for Mito-SOX, Mito-Tracker, BODIPY 581/591, FerroOrange and DHE Staining in vivo or in vitro according to the manufacturers’ instructions.

**Biochemical detection:** Serum amylase and LDH were completed in the automatic biochemical analyzer (Servicebio, Wuhan, CN) according to the biochemical assay kit instructions (C016-1 and A020-2, Nanjing Jiancheng Bioengineering Institute, CN).

**Enzyme-linked immunosorbent assay (ELISA):** LDH levels in cell supernate were detected by corresponding ELISA kits (SEB864Ra) from Cloud-Clone Corp, CN. Amylase levels in cell supernate were measured using corresponding ELISA kits (SEB454Ra) from Cloud-Clone Corp, CN. The mouse IL-6 ELISA kit (SEA079Mu, Cloud-Clone Corp USCN Life Science, Wuhan, China) and tumor necrosis factor-α (TNF-α) ELISA kit (SEA133Mu, Cloud-Clone Corp USCN Life Science, Wuhan, China) were used for the detection of the levels of IL-6 and TNF-α according to the manufacturer’s instructions. The absorbance (O.D.) was measured at 450 nm wavelength by enzyme-labeled instrument (Bio‐Rad, California, USA), and the sample concentration was calculated.

**Detection of antioxidant capability:** The total antioxidant capacity of pancreatic tissue or AR42J cells was measured by the ferric ion reducing antioxidant power (FRAP) method using a T-AOC Assay Kit (S0116, Beyotime, China) according to the manufacturer’s protocol. Briefly, for cell samples: ~1 million cells in 200μL cold PBS, homogenized/sonicated to release antioxidants, centrifuged at 12,000g for 5min at 4°C; supernatant retained. Total antioxidant capacity assay: 180μL FRAP working solution per 96-well. Add 5μL PBS (blank), 5μL FeSO₄ standards (standard curve), or 5μL sample + 0.15-1.5mM Trolox (positive control). Incubate at 37°C for 3-5min, measure A593, and calculate via standard curve.

**Western Blot Analysis:** Pancreatic tissues were lysed in cold RIPA (P0013B, Beyotime, CN). Protein (30 μg/lane) was denatured (95°C, 10 min), separated by 10% SDS-PAGE (80 V stacking, 120 V resolving), then transferred to PVDF membranes (200 mA, 90 min, 4°C). Membranes were blocked (5% milk/TBST, 1 h, RT), incubated with primary antibodies (4°C, overnight), washed (TBST, 3×10 min), then with HRP-secondary antibodies (1:5000, 1 h, RT). Bands were developed using Digital gel image analysis system (Bio‐Rad, California, USA) and the gray values of the bands were quantitatively analyzed by Image J software. The antibodies used in this study are listed in the Supplementary Table below.

**Co-immunoprecipitation (Co-IP):** We conducted the Co-IP experiment using the immunoprecipitation kit produced by Beyotime Ltd (P2179M). In short, AR42J cells were washed three times with pre-cooled PBS, then added pre-cooled RIPA lysis buffer and incubated on ice for 30 minutes (with 1 spin every 10 minutes); the lysates were scraped off, centrifuged at 12000 × g for 20 minutes, and the supernatant was collected; the protein concentration was determined by BCA method and adjusted to 1 μg/μL for storage. Add 500 μL protein solution and 20 μL Protein A/G beads to a tube, shake at 4°C for 1 hour to pre-clear, then centrifuge at 3000 rpm for 5 minutes to obtain the supernatant; add 1 μg anti-MFG-E8 antibody or 1 μg normal IgG, shake at 4°C for 12 hours for incubation. Add 30 μL of Protein A/G beads, incubate at 4°C for 2 hours, centrifuge at 3000 rpm for 5 minutes and discard the supernatant; rinse with RIPA buffer 3 times at room temperature. Finally, elution and western blot verification were carried out, along with ImageJ quantitative analysis.

**Statistical Analysis:** Data were analyzed using GraphPad Prism 10.1.2 Software (San Diego, California, USA) and expressed as means ± standard error (SEM). The t-test or one-way ANOVA and compared using Student Newman Keuls test was used to analyze the differences between groups. All experiments were conducted in triplicate. A P-value < 0.05 represented a significant difference.

**Supplementary Table 1: Characteristics of patients with acute pancreatitis.**

| **Characteristics** | **N (%) or mean ± SD** |  |
| --- | --- | --- |
| Number | 85 |  |
| Age (Year) | 48 ± 14.3 |  |
| Sex (male/female)  Body Mass Index | 53/32  22.0 ± 1.9 |  |
| *Classification*  Mild  Moderately severe  Severe | 51 (60.0%)  16 (18.8%)  18 (21.2%) |  |
| *Etiology* |  |  |
| Biliary | 34 (40.0%) |  |
| Alcoholic | 2 (2.4%) |  |
| Hypertriglyceremic | 22 (25.9%) |  |
| Others | 27 (31.7%) |  |
| *Treatments* |  |  |
| Conservative therapy | 61 (71.8%) |  |
| Percutaneous drainage | 6 (7.1%) |  |
| Laparotomy | 18 (21.1%) |  |
| APACHE II scores | 5.8 ± 5 |  |
| SOFA scores | 2.1 ± 3 |  |
| Local complication (Yes/No) | 21/64 |  |
| Organ failure (Yes/No) | 13/72 |  |
| Serum PCT (ng/ml) | 2.8 ± 6.4 |  |
| Serum CRP (mg/L) | 135.5 ± 166 |  |
| Serum lipase (U/L) | 1752.8 ± 1831 |  |
| Serum amylase (U/L) | 713.3 ± 963.7 |  |
| Blood Glucose(mmol/L)  HbA1c (%)  WBC(*10^9/L)  Serum creatinine(μmol/L)  Serum BUN (mmol/L)  HCT (%)  Serum calcium(mmol/L) | 8.5 ± 7.3  5.2 ± 2.1  11.6 ± 5.9  87.7 ± 99  7.3 ± 91  39 ± 11.5  2.1 ± 0.2 |  |

**Supplementary Table 2: Antibodies**

| **Antibody** | **Item No** | **Company and location** |
| --- | --- | --- |
| β-Actin Mouse Monoclonal Antibody | AF0003 | Beyotime Biotechnology, CN |
| SQSTM1/p62 Antibody | 5114 | Cell Signaling Technology, Beverly, MA, USA |
| FTH1 (D1D4) Rabbit mAb | 4393 | Cell Signaling Technology, Beverly, MA, USA |
| SLC7A11 Rabbit Polyclonal Antibody | AF7992 | Beyotime Biotechnology, CN |
| LC3B Antibody | 2775 | Cell Signaling Technology, Beverly, MA, USA |
| Cox2 (D5H5) Rabbit mAb (PTGS2) | 73315 | Cell Signaling Technology, Beverly, MA, USA |
| GPX4 (E5Y8K) Rabbit mAb | 59735 | Cell Signaling Technology, Beverly, MA, USA |
| PINK1 Rabbit Polyclonal Antibody | Af7755 | Beyotime Biotechnology, CN |
| MFG-E8 Antibody | sc-271574 | Santa Cruz Biotechnology, Inc. |
| ABCE1 Antibody | sc-518185 | Santa Cruz Biotechnology, Inc. |
| Normal Mouse IgG | A7028 | Beyotime Biotechnology, CN |
| Myeloperoxidase Rabbit mAb | 15178 | Cell Signaling Technology, Beverly, MA, USA |
| FIS1 (E3K9O) Rabbit mAb | 32525 | Cell Signaling Technology, Beverly, MA, USA |
| PGC1α Rabbit Polyclonal Antibody | AF7736 | Beyotime Biotechnology, CN |
| DRP1 (D6C7) Rabbit mAb | 8570 | Cell Signaling Technology, Beverly, MA, USA |
| TFAM Rabbit Polyclonal Antibody | AF8127 | Beyotime Biotechnology, CN |
| Goat anti-Mouse IgG antibody | 31430 | PIONEER Biotechnology, CN |
| Goat anti-Rabbit IgG antibody | 31460 | PIONEER Biotechnology, CN |
| MFN2 Rabbit Polyclonal Antibody | AF7473 | Beyotime Biotechnology, CN |

**Supplementary figure 1. The *mfge8* gene knockout mice were created.** Western blot analysis of the MFG-E8 expression level in the pancreas. n = 6, error bars indicate the SEM; * P<0.05. MFG-E8: milk fat globule - epidermal growth factor 8; WT, wild type; KO, knock out.

**
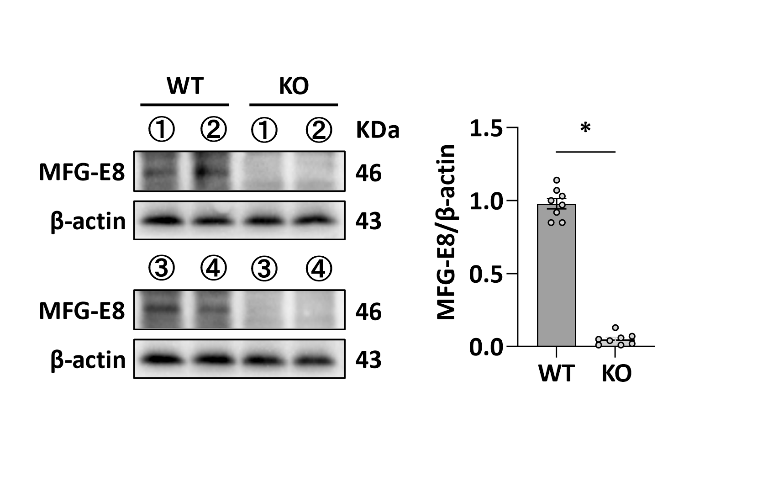
**

**Supplementary figure 2. Construct an AR42J cell line with *Mfge8* overexpression and ABCE1-KO dual gene modification** Western blot analysis of the MFG-E8 and ABCE1 expression level in AR42J. n = 12, error bars indicate the SEM; * P<0.05. MFG-E8: milk fat globule - epidermal growth factor 8; ABCE1: ATP - binding cassette subfamily E member 1; Lv, lentivirus; KO, knock out.

**
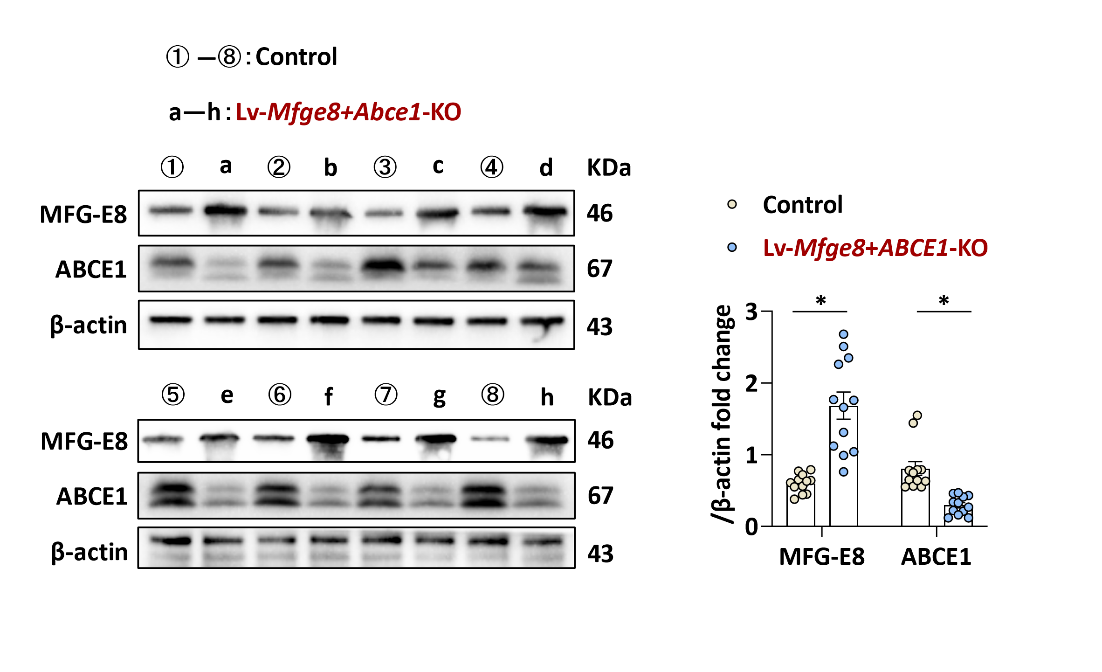
**

**Supplementary figure 3. Inhibition of ferroptosis antagonized the aggravating effect of MFG-E8-KO on experimental AP.** (**A**) Representative images of H&E staining of the pancreas (200X); (**B**) Pancreatic injury scores. n = 6, error bars indicate the SEM; * P<0.05. LPS, lipopolysaccharide; WT, Wild-type; KO: knockout; Fer-1: Ferrostatin-1; AP, acute pancreatitis.

**
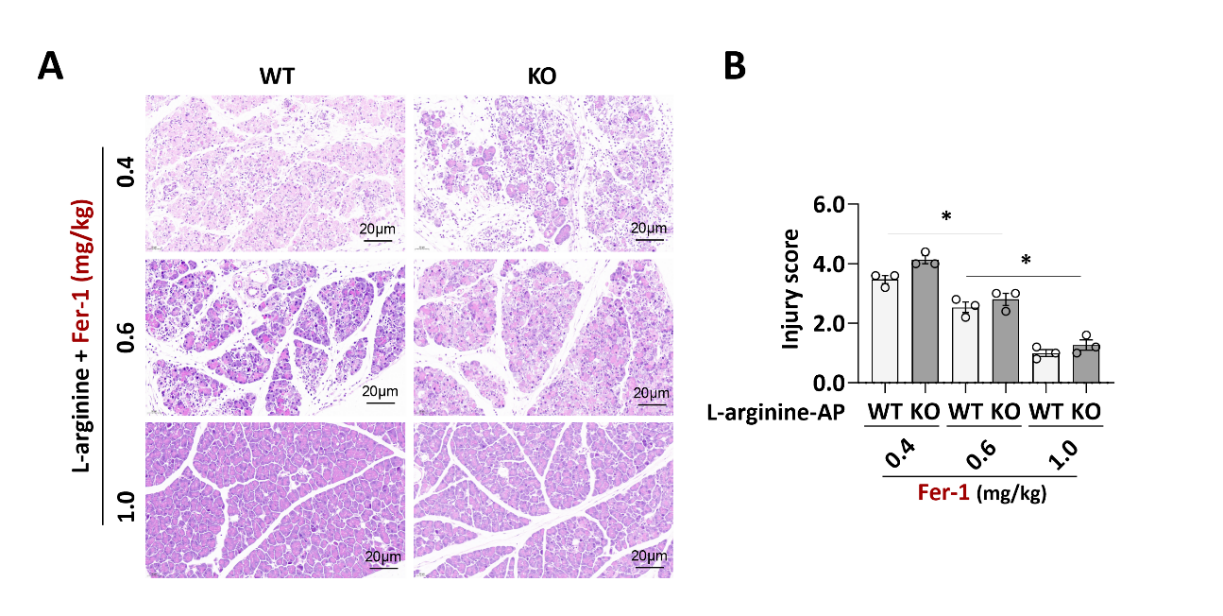
**

**Supplementary figure 4. Ferroptosis inhibition have no effect on the expression level of MFG-E8.** Western blot analysis of the MFG-E8 expression level in the pancreas. n = 4, error bars indicate the SEM; N.S., no significant differences. MFG-E8, milk fat globule - epidermal growth factor 8; Fer-1: Ferrostatin-1; WT, Wild-type; KO, knock out.

**
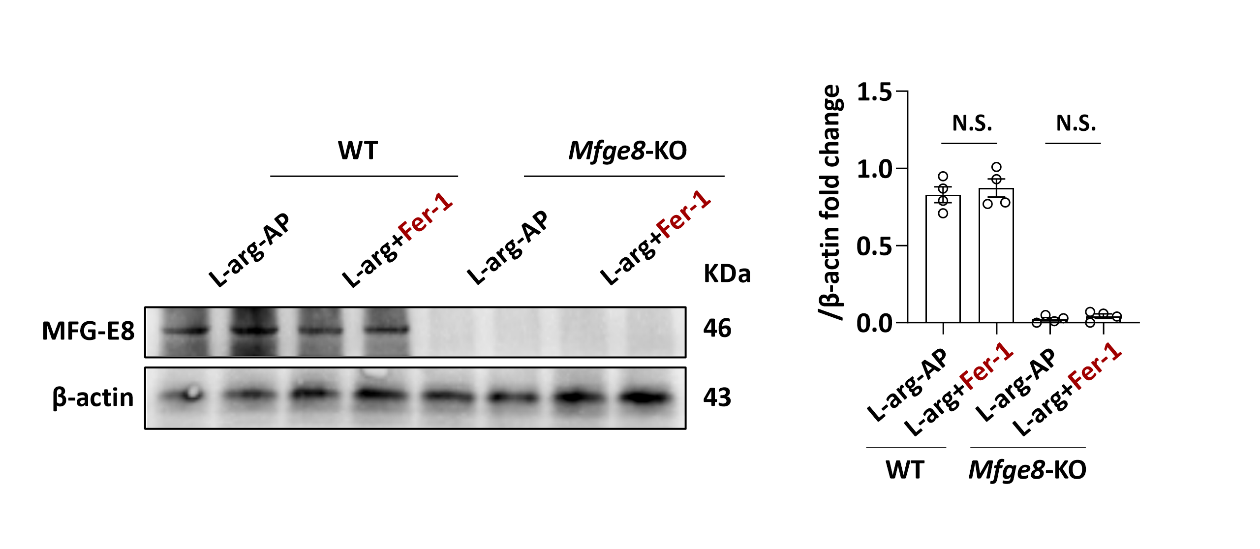
**

**Supplementary figure 5.** Entire membranes of the representative Western blot in Figure 1.

**
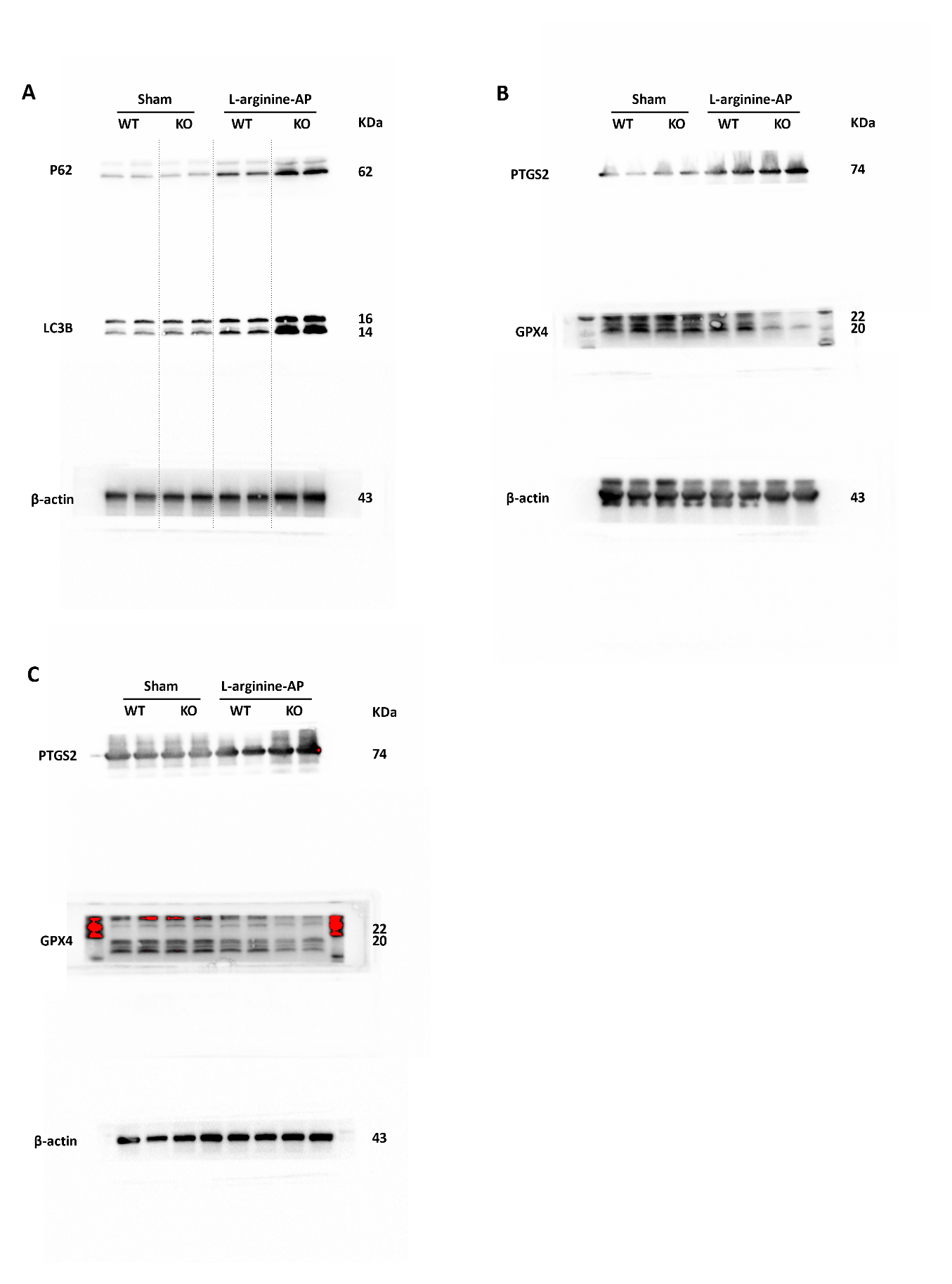
**

**Supplementary figure 6.** Entire membranes of the representative Western blot in Figure 2.

**
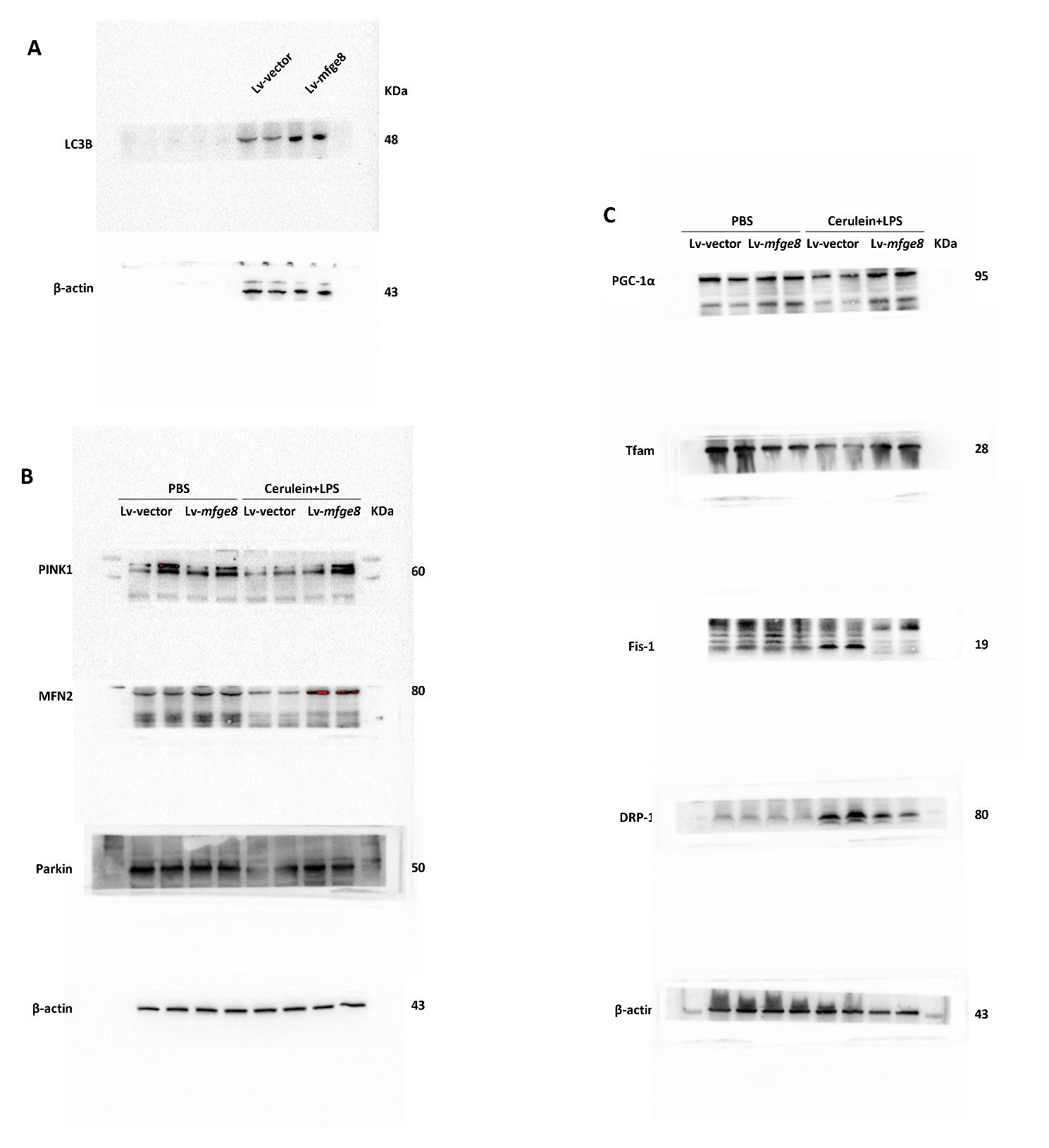
**

**Supplementary figure 7.** Entire membranes of the representative Western blot in Figure 3.

**
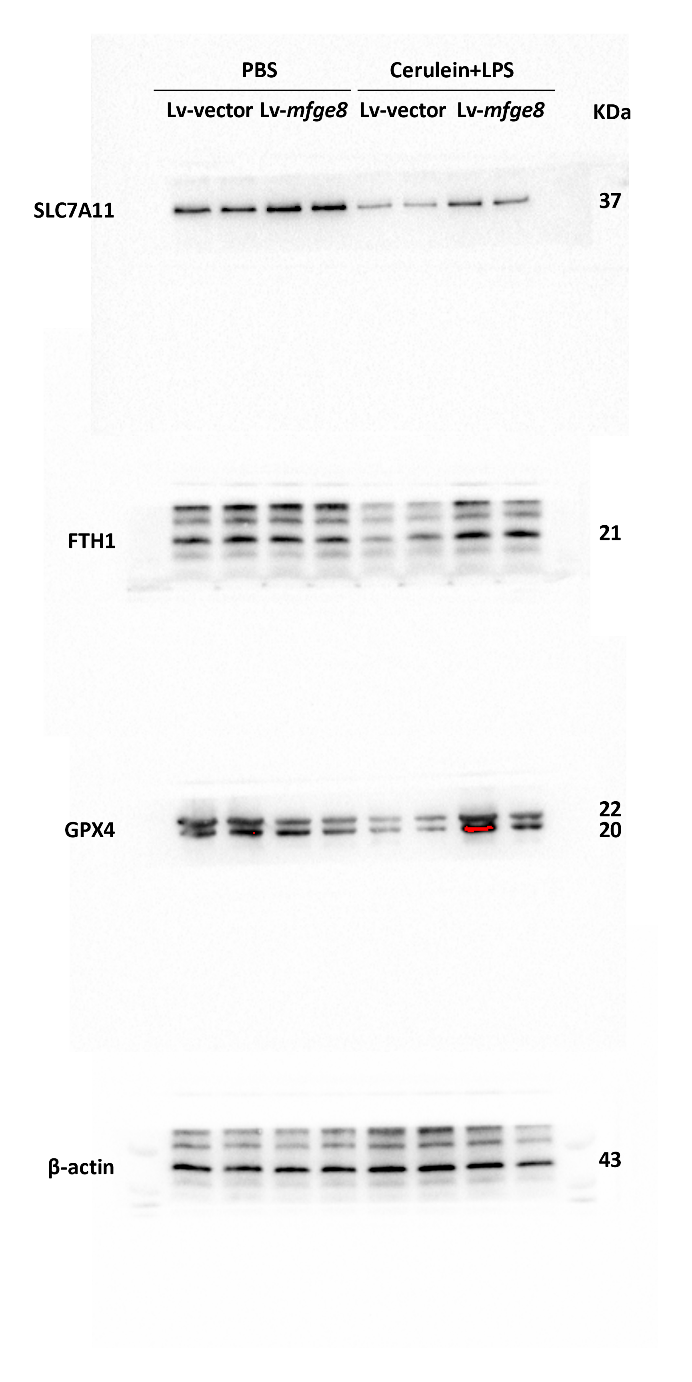
**

**Supplementary figure 8.** Entire membranes of the representative Western blot in Figure 4.

**
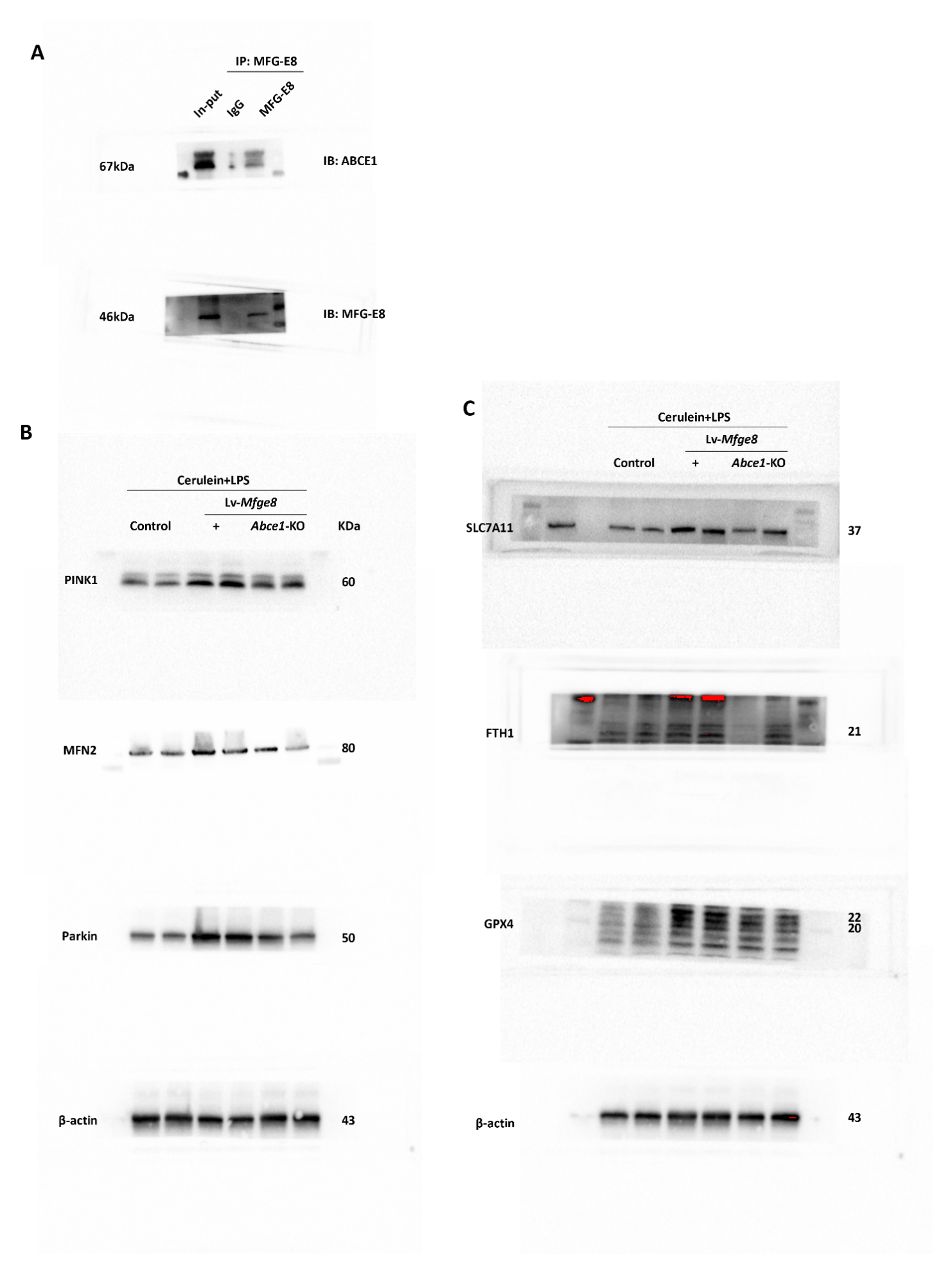
**

**Supplementary figure 9.** Entire membranes of the representative Western blot in Figure 5.

**
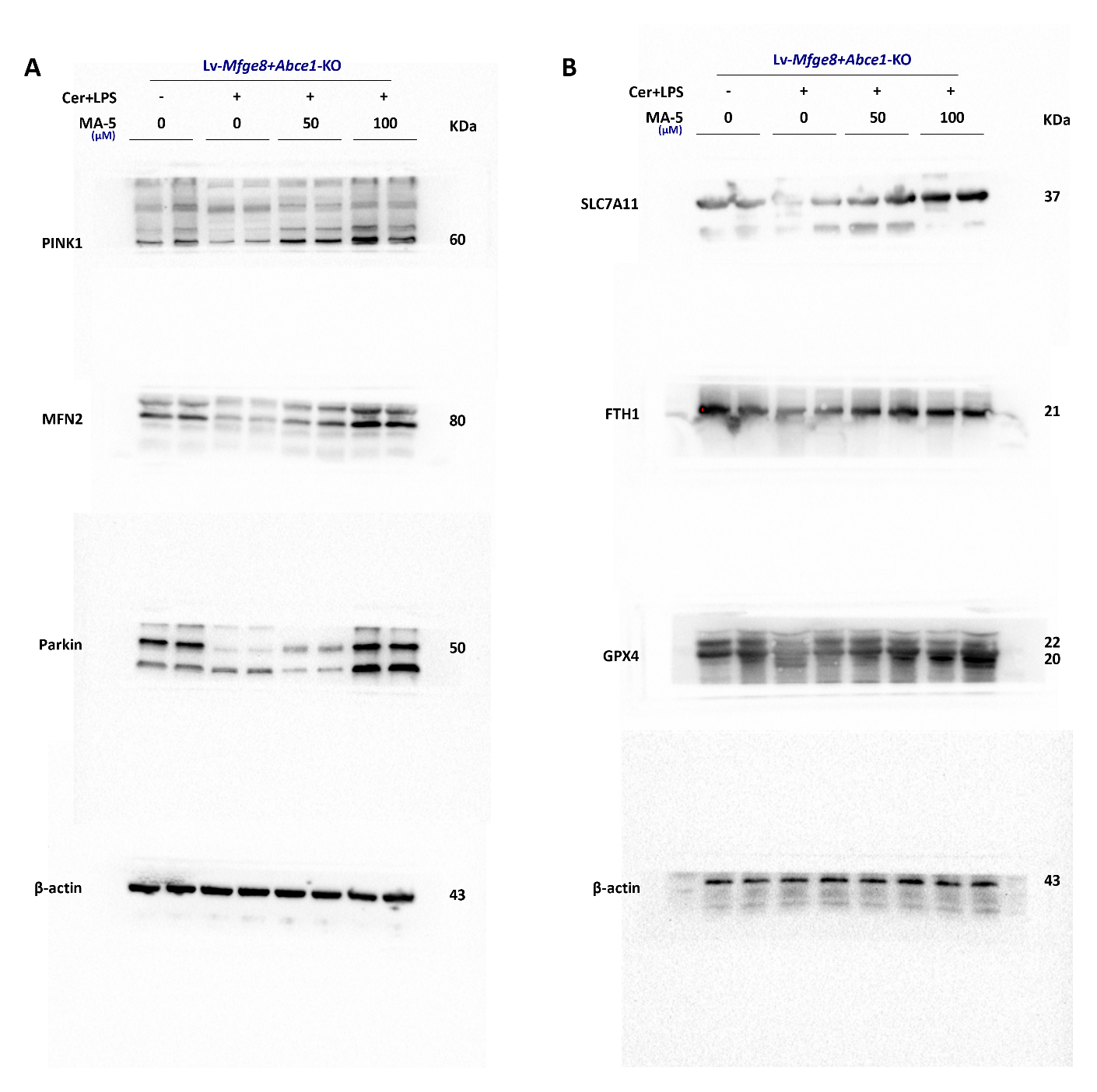
**

**Supplementary figure 10.** Entire membranes of the representative Western blot in Figure 6.

**
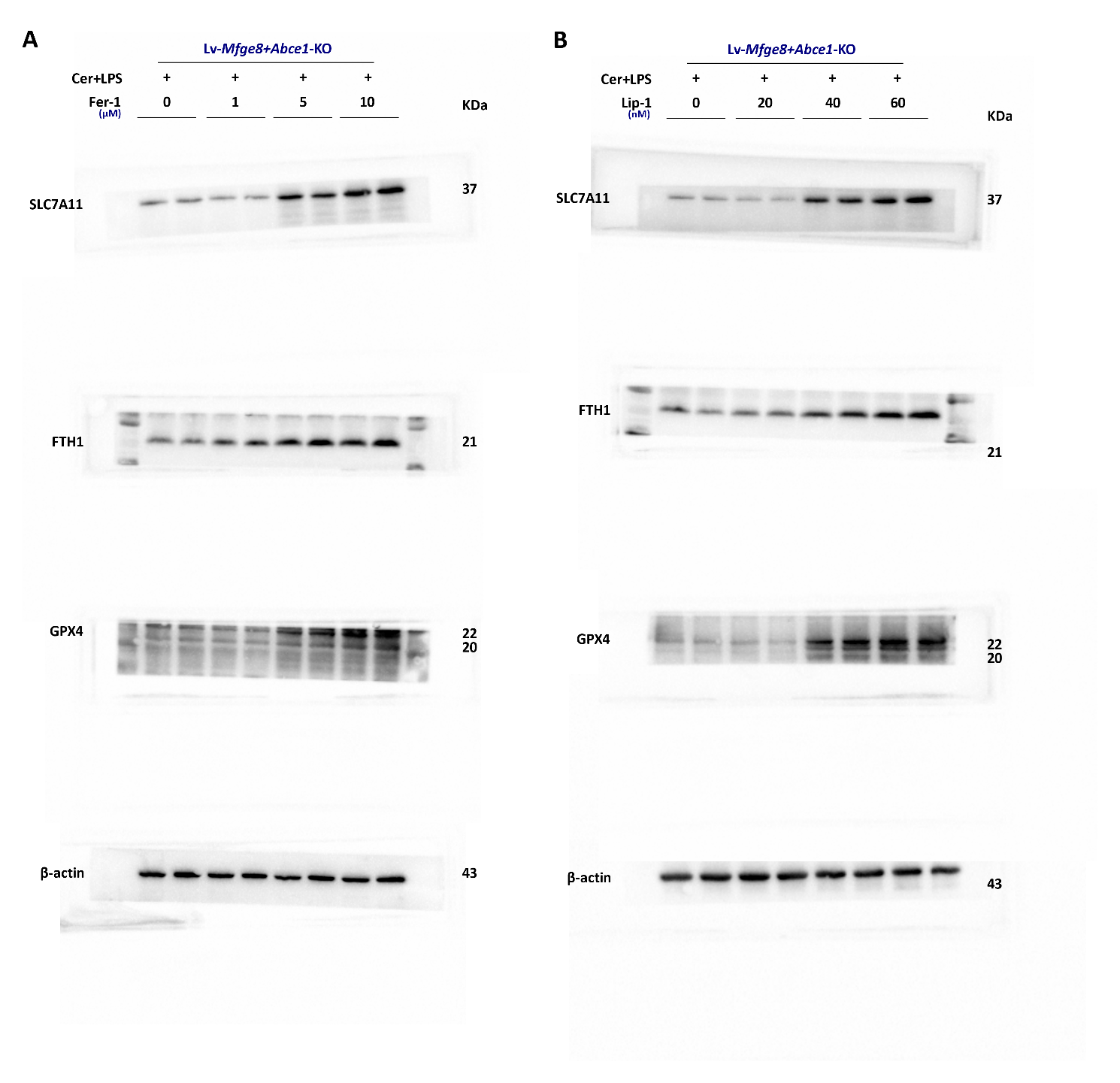
**

**Supplementary figure 11.** Entire membranes of the representative Western blot in Figure 7.

**
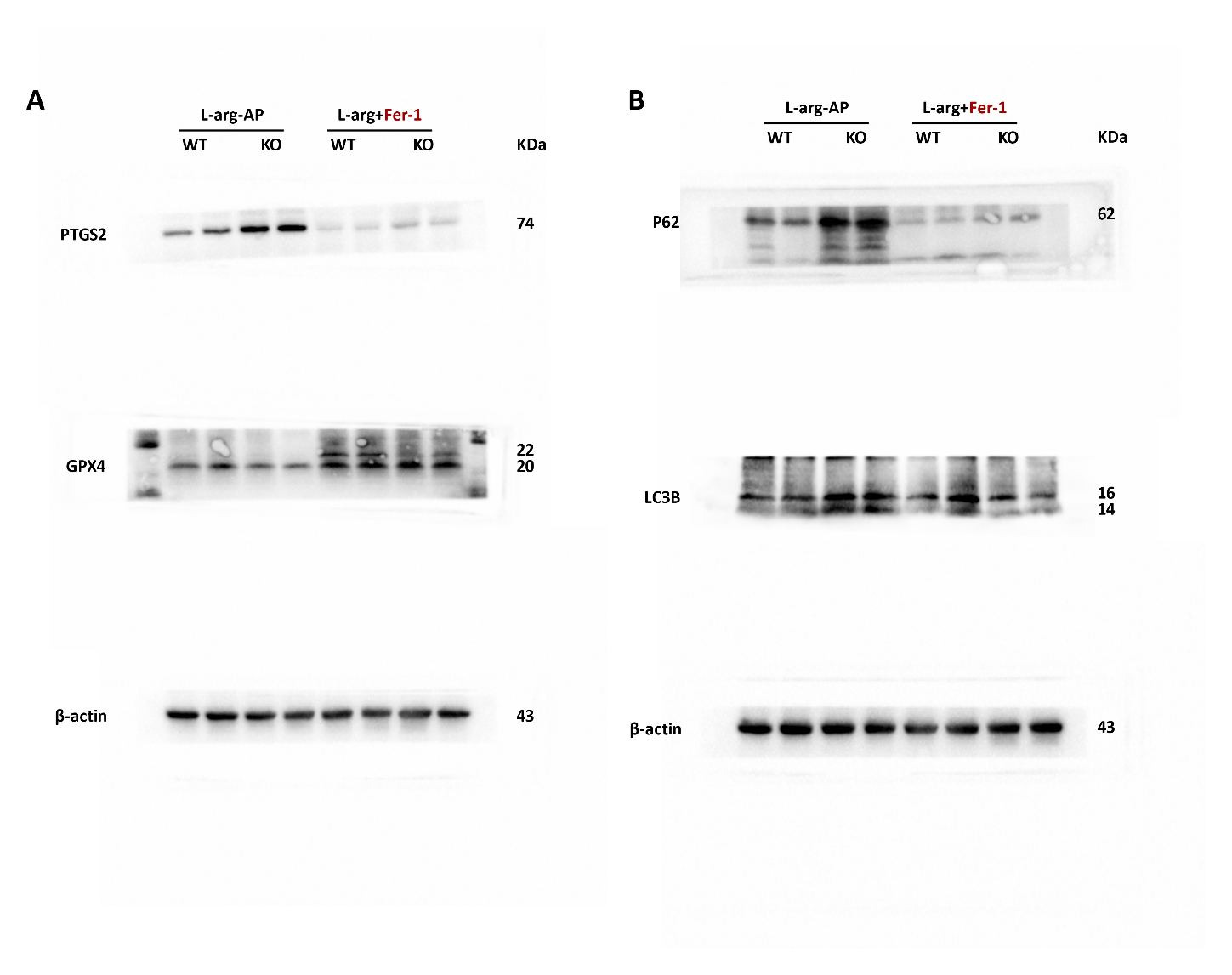
**

**Supplementary figure 12.** Entire membranes of the representative Western blot in Figure 8.

**
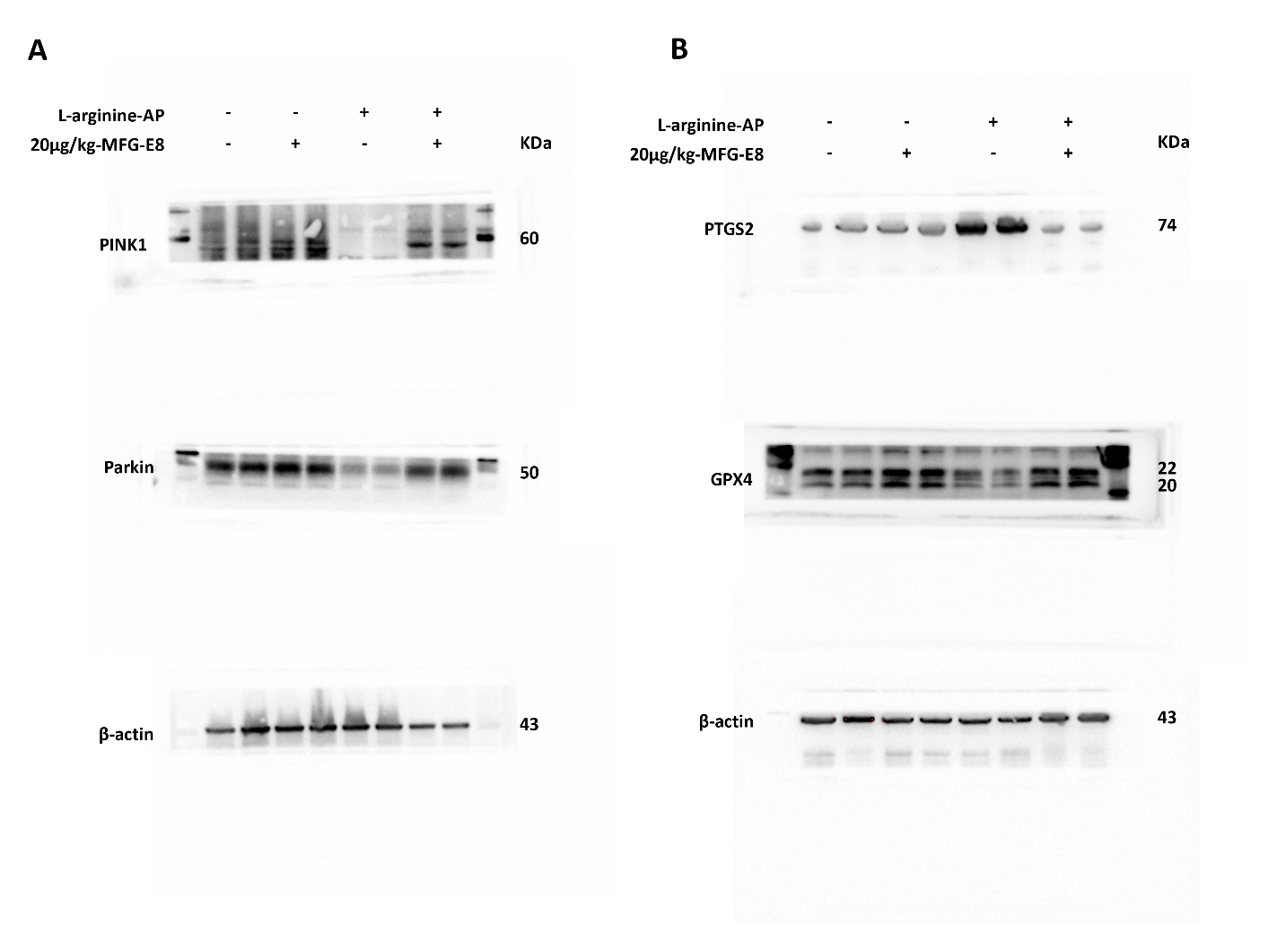
**

**Supplementary figure 13.** Entire membranes of the representative Western blot in Figure S1.

**
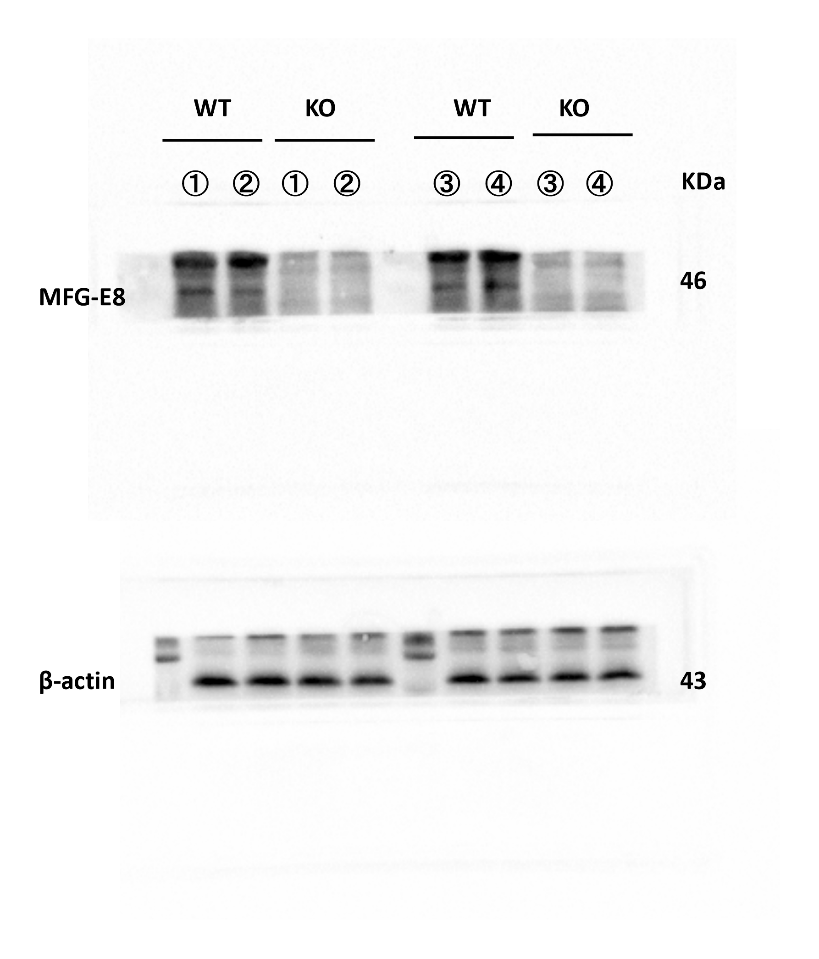
**

**Supplementary figure 14.** Entire membranes of the representative Western blot in Figure S2.

**
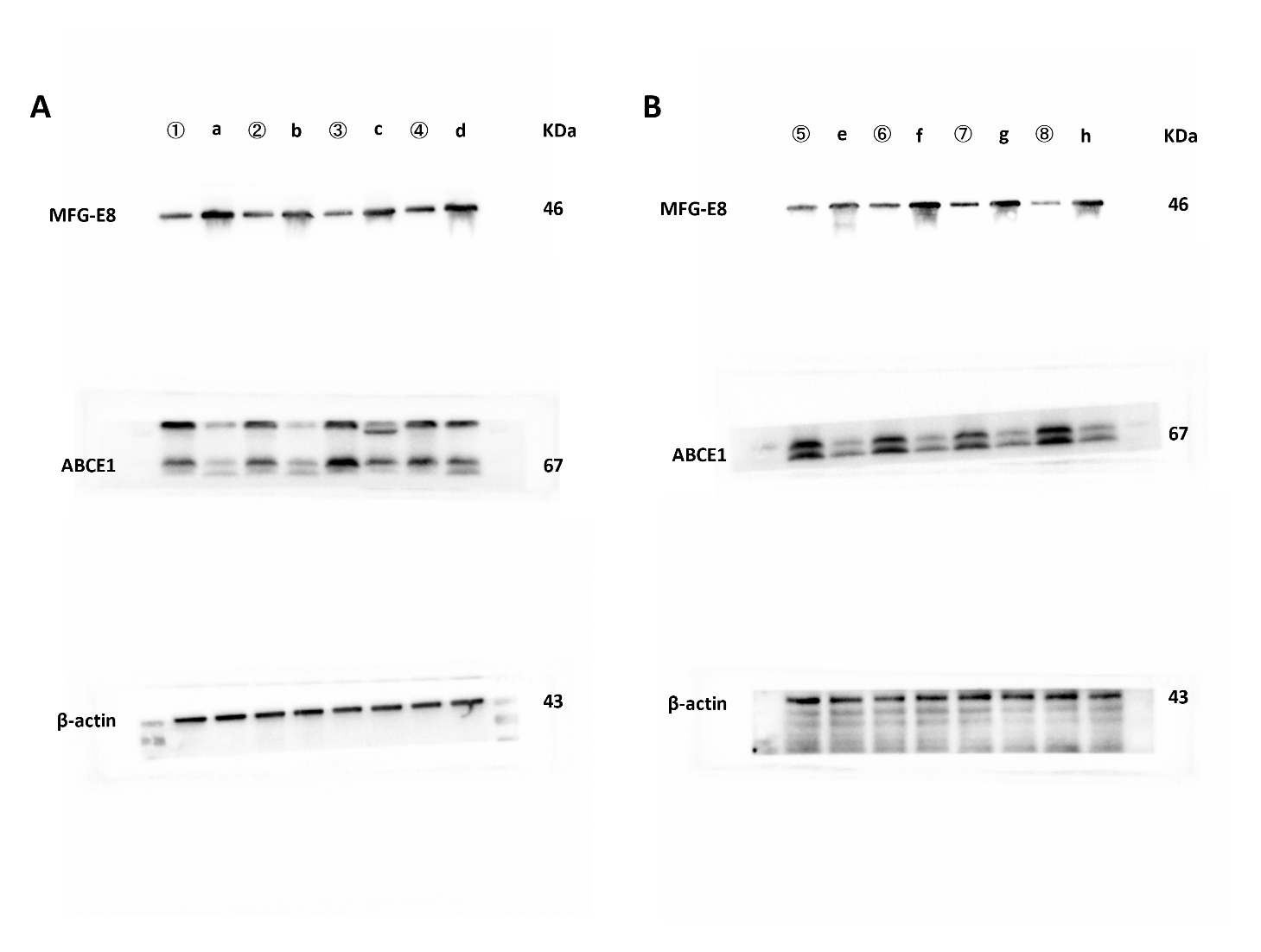
**

**Supplementary figure 15.** Entire membranes of the representative Western blot in Figure S4.

**
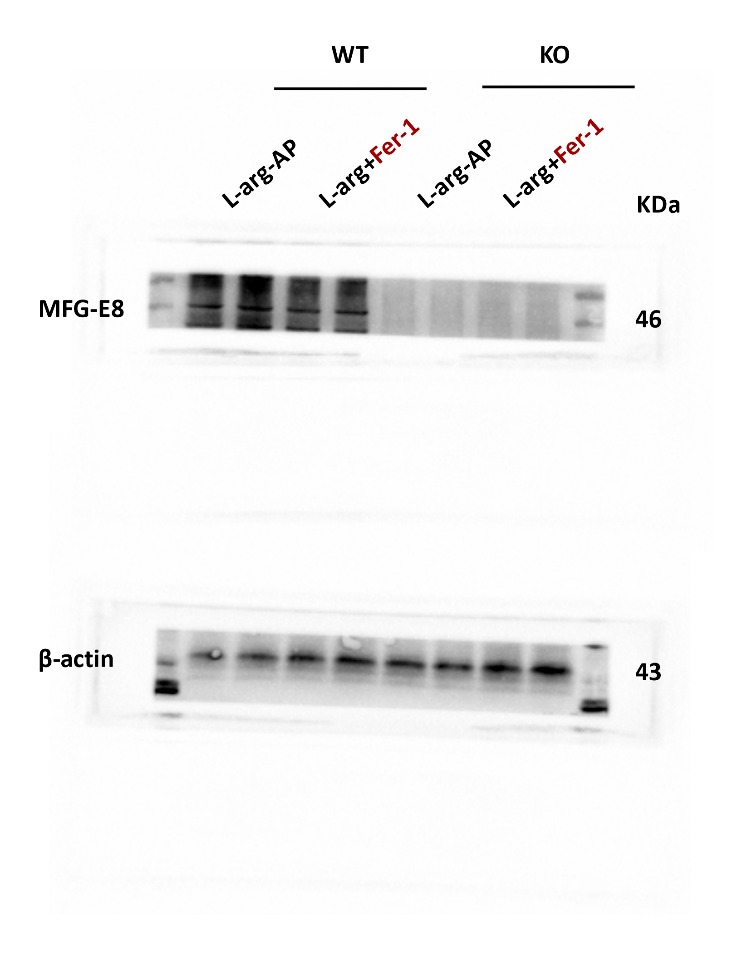
**

**Report on the Construction of Stable Cell Lines**

1. **项目信息**

**协议编号：STSOE241030CRT2**

**细胞名称：AR42J**

**细胞来源：内部细胞**

1. **分组信息**

| **组别** | **质粒名称** | **病毒批号** |
| --- | --- | --- |
| **过表达组** | LV-Mfge8 | 134DA07 |
| **对照组** | 阴性对照病毒CON254 | 134B3D4 |

1. **实验结论**

| **3.1** | **细胞转染** |
| --- | --- |
|  | 各实验组病毒转导效果良好。 |
| **3.2** | **QPCR检测** |
|  | Mfge8基因在过表达组中过表达40310.78倍。 |

1. **细胞稳转**

| AR42J培养体系： | F12K + 20%FBS |
| --- | --- |
| 传代比例： | 1:3传代 |
| MOI： | 50 |
| 筛选药物及浓度： | Puro 1μg/mL  药筛结束后以0.5μg/mL浓度维持至稳转株冻存。 |
| 将各组细胞送检无菌检测，经检测，无菌及支原体均为阴性。 | |

**
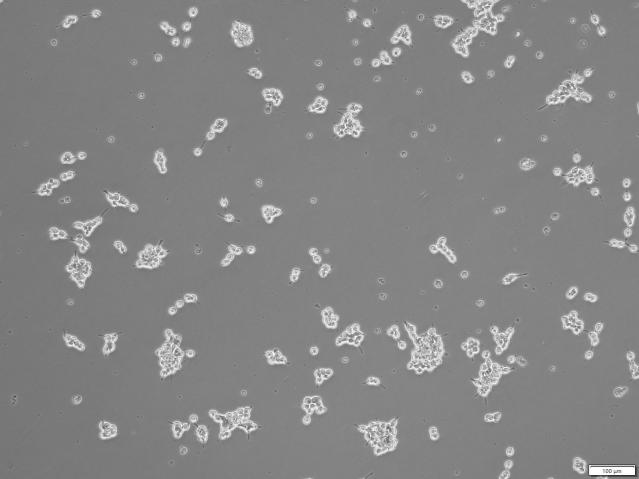

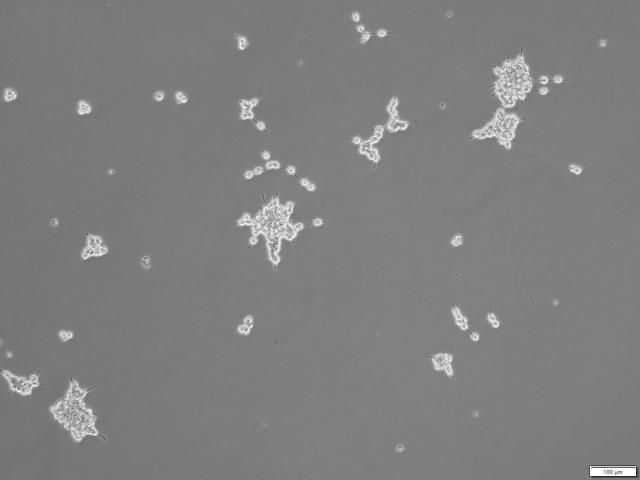
**

过表达组- Pn+4 -100x 对照组- Pn+4 -100x

1. **RT-qPCR**

**相对定量的方法检测目的基因的表达变化，按照2^-ΔΔCT^法对数据进行分析处理**


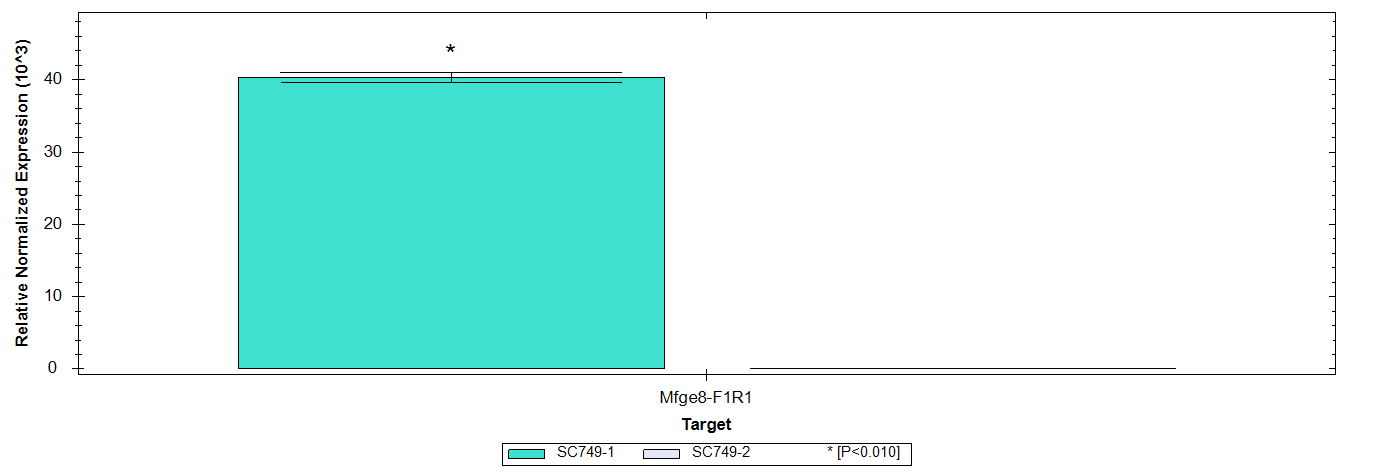


| 样品名称 | ① | ② | ΔCT  （①-②） | ΔΔCT  （实验组-对照组） | 2^-ΔΔCT^ |
| --- | --- | --- | --- | --- | --- |
|  | Mfge8 | rGAPDH |  |  |  |
| 过表达组 | 17.25±0.043 | 16.79±0.016 | 0.46 | -15.30 | 40310.78 |
| 对照组 | 32.92±0.936 | 17.16±0.032 | 15.76 | 0 | 1 |

**附录信息：**

| 基因名称 | 引物名称 | 引物序列 | 片段大小 |
| --- | --- | --- | --- |
| rGAPDH | rGAPDH-F | GATGCTGGTGCTGAGTATGT | 104 bp |
|  | rGAPDH-R | GCGGAGATGATGACCCTTT |  |
| Mfge8 | Mfge8-F | ATGGTGGGACCTGCTTGATG | 234 bp |
|  | Mfge8-R | TGGAACAGCCGAGTTCACAG |  |

**
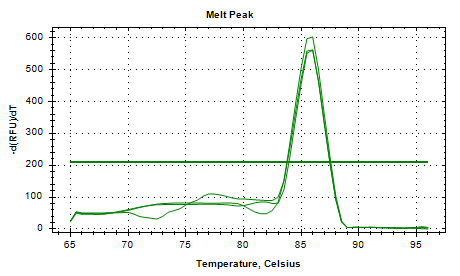
**

**Mfge8的溶解曲线**

**
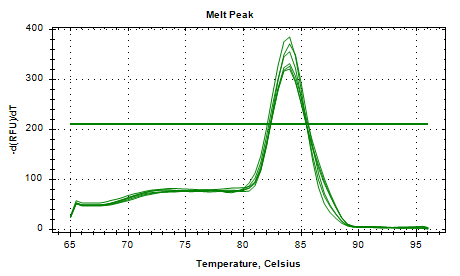
**

**rGAPDH的溶解曲线**

**Project report on overexpression of Mfge8 gene in Abce1 gene knockout AR42J monoclonal cells**

协议编号:：STSOE241030CRT1

项目编号：SC750

# 实验目的

构建Mfge8基因过表达Abce1基因敲除的AR42J细胞系。

# 方案设计

载体序列设计如下：

SC750-1：实验组Mfge8

# 实验结果汇总

| 序号 | 检测项 | 实验结论 | 附图 |
| --- | --- | --- | --- |
| 1 | qPCR鉴定 | qPCR鉴定目的基因过表达增高 | 图1、图2 |
| 2 | 细胞无菌检测和形态特征 | 无菌和支原体检测阴性；细胞形态正常。 | / |

# 实验信息

- 1. 关键仪器信息

| 仪器名称 | 品牌 | 型号 |
| --- | --- | --- |
| 生物安全柜 | Heal Force | HFsafe1200LC |
| CO_2_ 培养箱 | Heal Force | HF240 |
| 冰箱（-80℃冰箱） | 海尔 | DW-86L728 |
| 水浴锅 | 恒奥 | HWT-6B |
| qPCR仪 | Bio-Rad | CFX96 Touch |
| 自动细胞计数仪 | 上海睿钰 | IC 1000 |
| 细胞存活率分析仪 | BECKMAN | VI-CELL XR |

- 1. 关键实验试剂

| 试剂名称 | 品牌 | 货号 |
| --- | --- | --- |
| 培养基 | OriCell® | / |
| 胰酶 | OriCell® | TEDTA-10001 |
| 血清 | OriCell® | FBSST-01033 |
| DMSO | WAK-CHemie | 0482 |
| Puromycin | InvivoGen | ant-pr-1 |

# 实验步骤

分别使用实验组和对照组的慢病毒感染目的细胞，然后通过药物筛选细胞；将药筛后的细胞进行检测。

将包装好的慢病毒以MOI：50加入至提前铺好的目的细胞的24孔板中，重复混匀，细胞感染24h后撤去病毒，48h后加入筛选药物1μg/mLPuromycin进行筛选，筛选结束后以：0.5μg/mLPuromycin维持培养并进行鉴定。

# qPCR鉴定

采用相对定量的方法检测目的基因的表达变化，按照2^-ΔΔCT^法对数据进行分析处理；结果如下


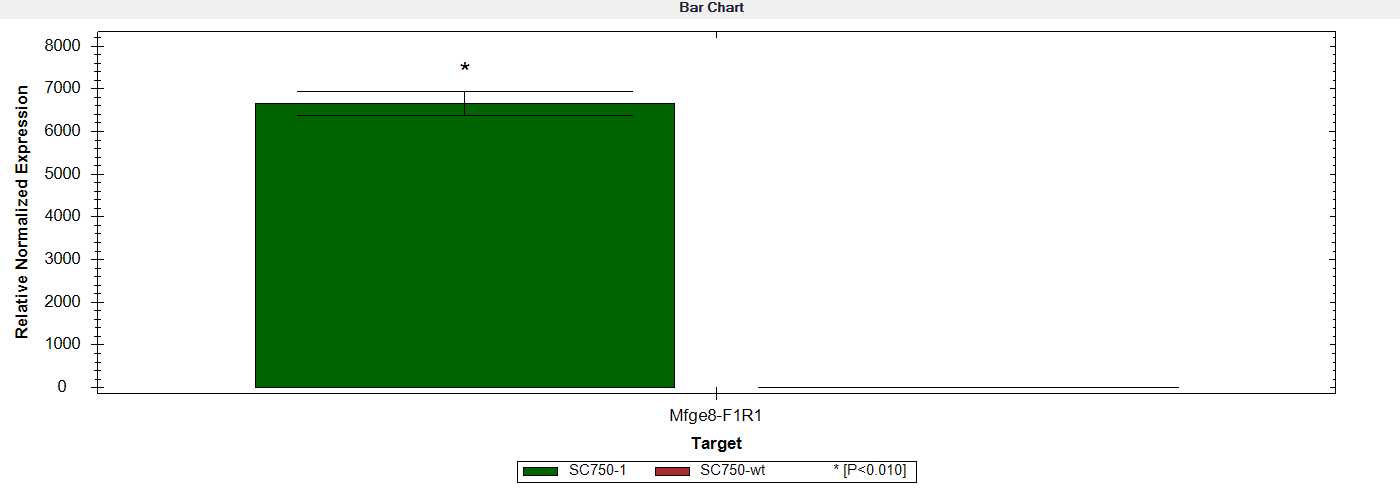


**图1 qPCR鉴定结果**

| 样品名称 | ① | ② | ΔCT  （①-②） | ΔΔCT  （实验组-对照组） | 2^-ΔΔCT^ |
| --- | --- | --- | --- | --- | --- |
|  | Mfge8 | hGAPDH |  |  |  |
| 过表达组 | 20.56±0.102 | 19.11±0.027 | 1.45 | -12.70 | 6653.97 |
| 对照组 | 33.09±0.351 | 18.94±0.066 | 14.15 | 0 | 1 |

| 基因名称 | 引物名称 | 引物序列 | 片段大小 |
| --- | --- | --- | --- |
| hGAPDH | hGAPDH-F | GAGTCCACTGGCGTCTTCAC | 123 bp |
|  | hGAPDH-R | ATGGTTCACACCCATGACGA |  |
| Mfge8 | Mfge8-F | ATGGTGGGACCTGCTTGATG | 234 bp |
|  | Mfge8-R | TGGAACAGCCGAGTTCACAG |  |

**
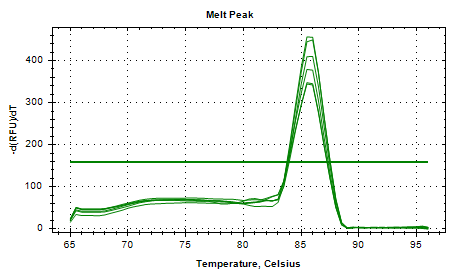

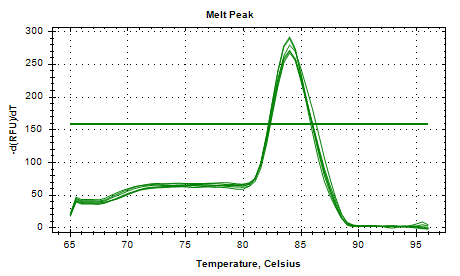
**

# 图2 Mfge8和hGAPDH的溶解曲线

# 细胞培养条件

| 细胞培养条件培养体系： | F12K+20%FBS培养基 |
| --- | --- |
| 传代比例： | 1:3传代 |
| 特殊培养细节： | ———— |
